# Both morph‐ and species‐dependent asymmetries affect reproductive barriers between heterostylous species

**DOI:** 10.1002/ece3.2293

**Published:** 2016-08-04

**Authors:** Barbara Keller, Jurriaan M. de Vos, Alexander N. Schmidt‐Lebuhn, James D. Thomson, Elena Conti

**Affiliations:** ^1^ Department of Systematic and Evolutionary BotanyUniversity of ZürichZollikerstrasse 1078008ZürichSwitzerland; ^2^ Department of Ecology and Evolutionary BiologyBrown University80 Waterman StreetBox G‐WProvidenceRhode Island02912USA; ^3^ CSIROAustralian National HerbariumGPO Box 1600CanberraAustralian Capital Territory2601Australia; ^4^ Ecology and Evolutionary Biology DepartmentUniversity of Toronto25 Harbord St.TorontoOntarioM5S 3G5Canada; ^5^ Present address: Comparative Plant and Fungal Biology DepartmentRoyal Botanic GardensKewRichmondSurreyTW9 3AE UK

**Keywords:** Diversification, floral heteromorphism, heterostyly, hybridization, mechanical isolation, morph‐dependent asymmetry, *Primula elatior* (oxlip), *Primula vulgaris* (primrose), reciprocal herkogamy, speciation, species‐dependent asymmetry

## Abstract

The interaction between floral traits and reproductive isolation is crucial to explaining the extraordinary diversity of angiosperms. Heterostyly, a complex floral polymorphism that optimizes outcrossing, evolved repeatedly and has been shown to accelerate diversification in primroses, yet its potential influence on isolating mechanisms remains unexplored. Furthermore, the relative contribution of pre‐ versus postmating barriers to reproductive isolation is still debated. No experimental study has yet evaluated the possible effects of heterostyly on pre‐ and postmating reproductive mechanisms. We quantify multiple reproductive barriers between the heterostylous *Primula elatior* (oxlip) and *P. vulgaris* (primrose), which readily hybridize when co‐occurring, and test whether traits of heterostyly contribute to reproductive barriers in unique ways. We find that premating isolation is key for both species, while postmating isolation is considerable only for *P. vulgaris*; ecogeographic isolation is crucial for both species, while phenological, seed developmental, and hybrid sterility barriers are also important in *P. vulgaris*, implicating sympatrically higher gene flow into *P. elatior*. We document for the first time that, in addition to the aforementioned species‐dependent asymmetries, morph‐dependent asymmetries affect reproductive barriers between heterostylous species. Indeed, the interspecific decrease of reciprocity between high sexual organs of complementary floral morphs limits interspecific pollen transfer from anthers of short‐styled flowers to stigmas of long‐styled flowers, while higher reciprocity between low sexual organs favors introgression over isolation from anthers of long‐styled flowers to stigmas of short‐styled flowers. Finally, intramorph incompatibility persists across species boundaries, but is weakened in long‐styled flowers of *P. elatior*, opening a possible backdoor to gene flow through intramorph pollen transfer between species. Therefore, patterns of gene flow across species boundaries are likely affected by floral morph composition of adjacent populations. To summarize, our study highlights the general importance of premating isolation and newly illustrates that both morph‐ and species‐dependent asymmetries shape boundaries between heterostylous species.

## Introduction

The interaction between floral traits and reproductive isolation is crucial to explaining angiosperm diversity. Flowers enable the evolution of complex relationships with pollinators, promoting reproductive isolation and diversification (Grant 1949). Specifically, attributes of corollas (*e.g.,* color, scent, texture, shape, tube length) and reproductive organs (*e.g.,* position, form, pollen/stigma ultrastructure and proteins) can facilitate isolating mechanisms by attracting different pollinators, restricting interspecific pollen transfer, or rejecting interspecific pollen (Lewis and Crowe 1958; Grant 1994; Schiestl and Schlüter 2009; Bomblies 2010). Heterostyly, a complex floral syndrome, has been shown to accelerate diversification in primroses (De Vos et al. 2014), yet no experimental study has evaluated its possible effects on reproductive isolation.

Reproductive barriers limit or prevent interspecific gene flow, maintaining species boundaries and increasing genetic distinctiveness between diverging lineages (Dobzhansky 1940; Mayr 1940). They are often classified into premating, postmating/prezygotic, and postzygotic barriers (Coyne and Orr 2004). Earlier‐acting barriers are thought to be more efficient, because they reduce the wastage of gametes and resources invested in the formation of potentially unfit hybrids (Ramsey et al. 2003). Natural selection should thus favor the evolution of earlier‐acting mechanisms, even when later‐acting ones exist (Butlin and Ritchie 2013). However, because premating barriers are affected by extrinsic, environmental factors, they are also considered to be more labile, hence postmating barriers may be necessary to ensure lasting reproductive isolation (Turelli et al. 2001; Coyne and Orr 2004; Seehausen et al. 2014). Indeed, while several studies found premating barriers to be stronger (Nosil et al. 2005; Martin and Willis 2007; Lowry et al. 2008; Sobel and Streisfeld 2015), others determined that postmating barriers are equally or more pronounced (Kozak et al. 2012; Scopece et al. 2013). Hence, the relative importance of pre‐ versus postmating barriers remains a key issue in evolutionary biology (Coyne and Orr 2004; Nosil 2012).

Specific morphological features of organs implicated in reproduction can contribute to premating isolation by mechanically limiting gamete exchange between species (*i.e.,* mechanical isolation; Coyne and Orr 2004; Butlin 2011). In animals, interspecific differences in body size or genital structure can prevent spatial or morphological matching of sexual organs (*e.g.,* damselflies; Sánchez‐Guillén et al. 2012, 2014). In angiosperms, anther and stigma positions can restrict pollen transfer to and collection from different body parts of shared pollinators (Grant 1949, 1994), limiting gamete wastage and pollen flow between species that occur sympatrically, flower at the same time, and share pollinators (Coyne and Orr 2004). However, conclusive experimental evidence of mechanical isolation is rare in both animals (Masly 2012) and plants (Campbell and Aldridge 2006).

Assessing mechanical isolation in angiosperms requires quantitative comparisons between inter‐ and intraspecific pollen transfer in relation to specific reproductive features (Campbell and Aldridge 2006). Thus, mechanical barriers are rarely measured directly, because precise pollen‐grain counts are difficult to acquire (Campbell et al. 1998; Wolf et al. 2001; Muchhala and Potts 2007; Natalis and Wesselingh 2012). Mechanical isolation has also been indirectly inferred using pollen analogs (Kay 2006; Brock 2009; Martin and Taylor 2013), pollen placement on pollinator's bodies (*e.g.,* Nilsson 1983; Kephart and Theiss 2003; Sun et al. 2011), and differential positions of anthers and stigmas in hybridizing species (Yang et al. 2007; Keller et al. 2012). Complete mechanical isolation has been conclusively demonstrated only for *Costus pulverulentus* (Kay 2006).

Because premating barriers are usually insufficient to interrupt interspecific gene flow, postmating barriers are necessary for complete reproductive isolation (Widmer et al. 2008). The formation of viable hybrids may be prevented *via* different mechanisms, including negative egg–sperm and pollen–pistil interactions in animals and plants, respectively (Galindo et al. 2003; Swanson et al. 2004), dosage imbalances between parental genomes (*e.g.,* unbalanced development of endosperm versus zygote in plants; Feil and Berger 2007); genetic incompatibilities at specific loci of the maternal and paternal genomes (Bateson‐Dobzhansky‐Muller incompatibilities: BDM‐I's; Bateson 1909; Dobzhansky 1936; Muller 1942; Orr 1996), and chromosomal rearrangements (Stebbins 1950; Rieseberg et al. 1999; Noor et al. 2001). Finally, hybrids may fail to establish and reproduce when they are outcompeted by parental individuals or sterile, respectively (Campbell and Waser 2001; Widmer et al. 2008).

Reproductive barriers often act asymmetrically. Previously documented asymmetries depend on which species provides the female and male gametes, respectively, to hybrid formation (*i.e.,* species‐dependent asymmetries; Rieseberg and Carney 1998; Wirtz 1999; Tiffin et al. 2001; Turelli and Moyle 2007; Lowry et al. 2008; Arnold et al. 2010). In animals, the differential fit between male and female reproductive organs in the two cross‐directions may cause asymmetries in mechanical isolation (Sánchez‐Guillén et al. 2012). Similarly, in angiosperms, stigmas of one species may contact zones of the pollinator's body that carry heterospecific pollen, while stigmas of the other species may fail to do so, restricting interspecific gene flow in one direction (Wolf et al. 2001; Kay 2006). Species‐dependent asymmetries may also occur at the postmating, prezygotic stage. For instance, interspecific differences in pistil length and pollen compatibility (De Nettancourt 2001) may allow male gametes to reach and fertilize ovules, respectively, only in one cross‐direction (*e.g.,* Gore et al. 1990; Yost and Kay 2009). At the postzygotic stage, asymmetries may arise due to genetic incompatibilities that allow embryo development only in one cross‐direction (Turelli and Moyle 2007). For example, genomic imbalances can cause asynchronous growth of embryo and endosperm, generating stronger asymmetries of hybrid seed development in one cross‐direction than the other (*e.g.,* Valentine and Woodell 1960; Johnston et al. 1980). In addition to species‐dependent asymmetries, morph‐dependent asymmetries may exist in hermaphroditic species with heteromorphic individuals, although they have not yet been investigated.

**Figure 1:**
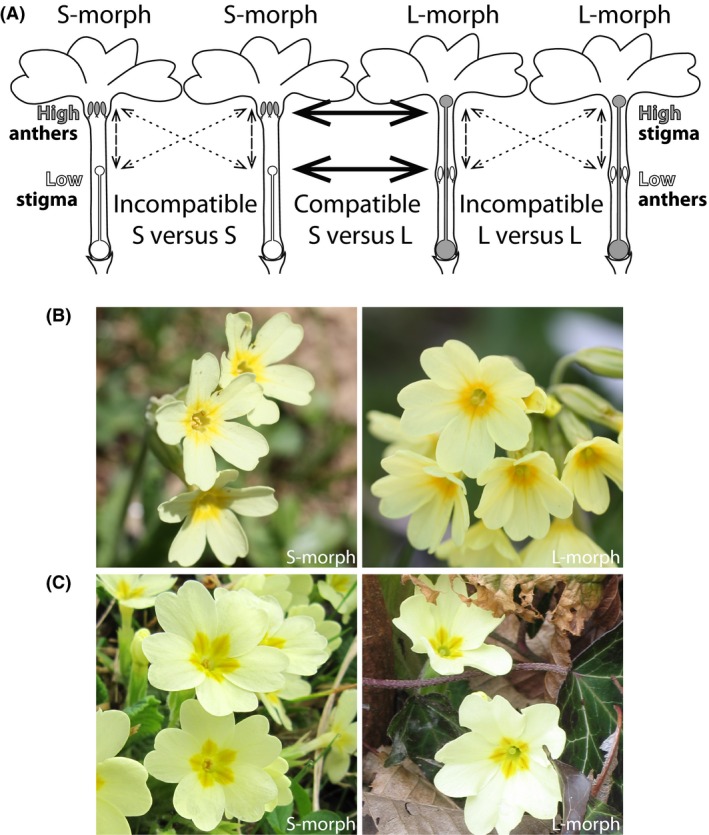
Heterostyly in *Primula*: (A) Diagrams of short‐styled (S‐) and long‐styled (L‐) morphs of distylous *Primula sp*., with sexual organs placed reciprocally at two levels in the corolla tubes of compatible, heteromorphic flowers (*i.e*., reciprocal herkogamy). Photographs of S‐ and L‐morphs of (B) *Primula elatior* and (C) *Primula vulgaris*. High anthers of S‐morphs match the position of high stigmas in L‐morphs, and low anthers of L‐morphs match the position of low stigmas in S‐morphs (*i.e*., sexual organ reciprocity between exposed and sunken organs, respectively; solid arrows). Conversely, incompatible sexual organs of the same flower or floral morph (*i.e*., homomorphic) do not match spatially (dashed and dotted arrows, respectively). Distyly promotes pollen transfer between heteromorphic, compatible flowers (*i.e*., disassortative pollination). Photograph (B) courtesy of Florian Boucher; the others were taken by the first author in natural Swiss populations.

A common type of heteromorphism in angiosperms is heterostyly, described in 119 genera of at least 28 families (Lloyd and Webb 1992; Barrett 2002; Naiki 2012). Heterostylous populations comprise two (distyly) or, more rarely, three (tristyly) genetically determined floral morphs differing in the reciprocal placement of sexual organs (*i.e.,* reciprocal herkogamy; Ganders 1979). In distylous flowers, high anthers of short‐styled morphs spatially match high stigmas of long‐styled morphs (hereafter, S‐ and L‐morph, respectively), while low anthers of L‐morphs match low stigmas of S‐morphs (*i.e*., sexual organ reciprocity). Conversely, sexual organs of the same flower or floral morph (*i.e*., homomorphic) do not match spatially (Fig. 1A). A sporophytic incompatibility system often ensures pollen rejection within the same flower or between flowers of the same morph (hereafter, “intramorph incompatibility”). Distyly promotes cross‐fertilization between compatible, heteromorphic flowers *via* the transfer of pollen onto distinct positions of the pollinator's body corresponding to the heights of the receiving stigmas (*i.e.,* disassortative pollination), decreasing gamete wastage to self‐fertilization and sexual interference (Barrett 2002). In the best known distylous system, *that is* primroses (*Primula* L.; Barrett and Shore 2008; Gilmartin and Li 2010), a single Mendelian, diallelic locus (*i.e.,* S‐locus) controls distyly, with L‐plants being homozygous (*ss*) and S‐plants heterozygous (*Ss*). This genetic system, coupled with disassortative mating between morphs, maintains equal morph ratios (*i.e.,* isoplethy) in sufficiently large populations (Dowrick 1956; Lewis and Jones 1992).

Distyly might influence reproductive isolation in complex ways. For example, intra‐ versus interspecific differences of sexual organ reciprocity might promote mechanical barriers. Within species, the closer spatial matching between reciprocal than nonreciprocal sexual organs is associated with greater heteromorphic than homomorphic pollen transfer (Fig. 1A; Lau and Bosque 2003; Baena‐Díaz et al. 2012; Keller et al. 2014; Zhou et al. 2015). Between species, a decrease of sexual organ reciprocity (as observed in *Primula*; Keller et al. 2012) might thus restrict interspecific pollen movement between reciprocal morphs, hypothetically contributing to mechanical isolation (Haller et al. 2014).

Furthermore, the occurrence of hermaphroditic morphs with placement of sexual organs at two levels might enable morph‐dependent asymmetries of reproductive barriers. Within species characterized by insect‐pollinated, tubular flowers, the high stigma of the L‐morph receives significantly more pollen than the low stigma of the S‐morph (Stone and Thomson 1994; Matsumura and Washitani 2002; Ornelas et al. 2004; Keller et al. 2014 for primroses: Fig. 1A). If the difference of pollen exchange between exposed and sunken organs is maintained interspecifically and the significantly lower number of ovules than pollen grains in angiosperms is considered (*e.g.,* in distylous primroses: Ornduff 1979; Schou 1983; Piper and Charlesworth 1986), selection to restrict access of interspecific pollen to ovules might be stronger on the L‐ than S‐morph, increasing mechanical isolation in the former over the latter, a prediction tested in this study.

Distylous species thus represent a unique system to investigate both mechanical isolation and the potential for morph‐dependent, besides species‐dependent, asymmetries in reproductive barriers. Additionally, morph‐dependent directionality of isolation might have far‐reaching eco‐evolutionary implications in cases of skewed morph ratios in distylous populations, which have been documented in *Primula* and other species (*e.g.,* Meeus et al. 2012). Nevertheless, the comparisons of inter‐ versus intraspecific pollen transfer necessary to empirically test the potential role of distyly in reproductive isolation have never been performed. More generally, detailed studies of sequential reproductive barriers are not available for heterostylous species, precluding new knowledge on how floral heteromorphism might shape angiosperm evolution.

The phylogenetically close *Primula elatior* (Fig. 1B) and *Primula vulgaris* (Fig. 1C) (Mast et al. 2006; Schmidt‐Lebuhn et al. 2012) represent an ideal species pair to elucidate the interaction between distyly and reproductive isolation, because they readily hybridize and backcross, forming hybrid swarms when co‐occurring (Valentine 1948; Woodell 1969; Gurney et al. 2007; Taylor and Woodell 2008; Jacquemyn et al. 2009; B. Keller, pers. obs.). Because primroses have been extensively researched since Darwin (1862, 1868, 1877), numerous studies are available on their distylous floral traits (*e.g.,* Fey 1929; Keller et al. 2012, 2014), ecological preferences (Valentine 1948; Woodell 1969; Taylor and Woodell 2008; Jacquemyn et al. 2009), and postmating reproductive barriers (De Vries 1919/20; Valentine 1947, 1948, 1953; Woodell 1960a). Finally, the degree of spatial matching between reciprocal sexual organs is lower between than within these two species (Keller et al. 2012), suggesting that distyly might contribute to mechanical isolation.

Despite the crucial role of pre‐ and postmating isolation in the processes that generate and maintain species diversity (*e.g.,* Nosil 2012), few detailed analyses of multiple reproductive barriers are available in plants (*e.g.,* Lowry et al. 2008; Scopece et al. 2013; Brys et al. 2014; Carrió and Güemes 2014; Melo et al. 2014; Sedeek et al. 2014), and none in heterostylous species. Even fewer studies focus on mechanical isolation (Wolf et al. 2001; Kay 2006; Chen 2011; Brys et al. 2014), and none has yet investigated whether heterostylous traits alter interspecific boundaries in distinctive, possibly asymmetric ways. In order to examine the specific contributions of heterostyly to reproductive isolation, we thus assess a series of pre‐ and postmating barriers between *P. elatior* and *P. vulgaris* at different stages of the life cycle, including ecogeographic characteristics, flowering phenology, and pollen transfer between parental species, as well as formation, survivorship, and reproduction of hybrids. We hypothesize that the decrease of sexual organ reciprocity documented between *P. elatior* and *P. vulgaris* (Keller et al. 2012) might restrict interspecific pollen movement, contributing to mechanical isolation. Finally, we expect that distyly might impose morph‐dependent, in addition to species‐dependent asymmetries on reproductive barriers. As explained above, mechanical isolation should be stronger for the L‐morph than the S‐morph. This study thus represents the first, in‐depth analysis of the special means by which heteromorphy in hermaphroditic flowers might modulate gene flow between species.

## Materials and Methods

### Study plants

*Primula elatior* Hill (oxlip) and *P. vulgaris* Huds. (primrose) are perennial, rosette‐forming diploids (*2n* = 22) with phenotypically similar distylous flowers characterized by pale‐yellow corollas with broad, v‐notched lobes, but differing in flower width, corolla limb and tube length, sexual organ height (all greater in *P. vulgaris* than *P. elatior*; Keller et al. 2012), and inflorescence structure (pedunculate scapes in *P. elatior*; pedicellate single flowers in *P. vulgaris*; Richards 2003). Both species have high degrees of reciprocal herkogamy and strong, but incomplete intramorph incompatibility (Ornduff 1979; Wedderburn and Richards 1990; Keller et al. 2012, 2014). Their F1 hybrids are morphologically intermediate to the parents, but backcrosses and later‐generation hybrids are usually indistinguishable from parental species (Gurney et al. 2007). To decrease the risk of including hybrids in our study, we used plants from a local wild‐plant nursery to quantify mechanical barriers and plants from allopatric Swiss populations to quantify postmating barriers.

**Figure 2:**
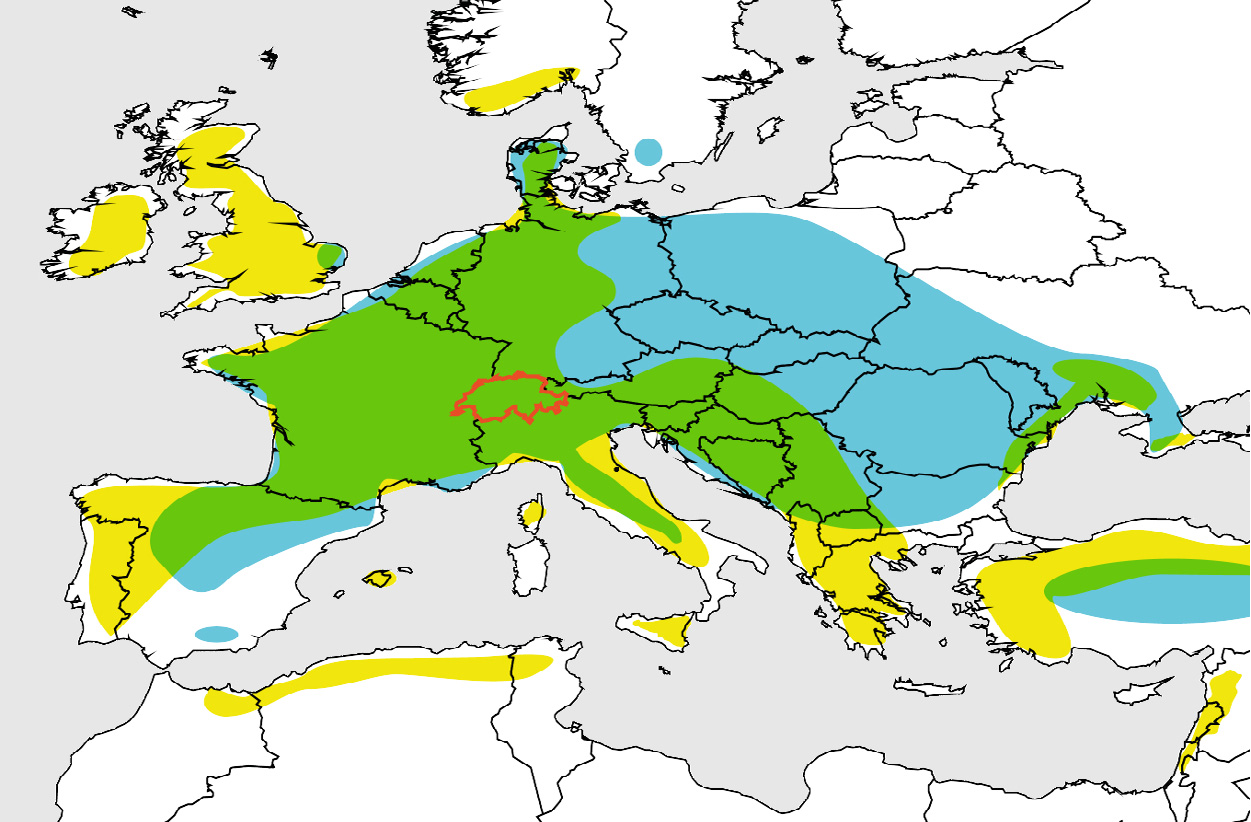
Distributional ranges of *Primula elatior* (blue), *Primula vulgaris* (yellow), and their overlap (green) in Europe. Distributional maps were generated from data compiled from Global Biodiversity Information Facility (GBIF: http://data.gbif.org/), Info Flora (www.infoflora.ch), Flora Web Deutschland (http://www.floraweb.de), Flora Europaea (Valentine and Kress 1972), Flora of the USSR (Komarov 1963), and Richards (2003, personal communication). Highlighted in red is Switzerland, where occurrences records of both species were used to estimate ecogeographic barriers (RI_ecogeo_) and the experimental part of our study was conducted (see text).

Widespread in Europe, *P. elatior* and *P. vulgaris* occur in both allopatric and sympatric populations within their largely overlapping distributional ranges (Taylor and Woodell 2008; Jacquemyn et al. 2009), including in Switzerland, where our study was conducted (B. Keller, pers. obs; Fig. 2). *Primula elatior* prefers moister habitats and tolerates colder winter/spring temperatures, spanning a broader altitudinal range than *P. vulgaris* (*e.g.,* Hegi 1935; Valentine 1948; Woodell 1969). Both species flower in spring (*P. elatior*: March–May; *P. vulgaris*: March–April [‐May]; Lauber and Wagner 2007; Taylor and Woodell 2008; Jacquemyn et al. 2009) and are visited by the same generalist insects (Christy 1922; Woodell 1960b; Richards 2003). Thus, ethological barriers are unlikely to contribute significantly to reproductive isolation.

### Reproductive isolation

We quantified the strengths of three pre‐ and six postmating barriers between *P. elatior* and *P. vulgaris* following the method by Sobel and Chen (2014), with barrier strengths (Reproductive isolation, RI‐values) ranging from one (complete isolation: no interspecific gene flow) through zero (no isolation: equal probability of intra‐ and interspecific gene flow) to minus one (no isolation: all gene flow is interspecific). Barrier strengths were calculated with means estimated by generalized mixed‐effects models (GLMMs) that account for relatedness and maternal effects of plants used in experimental crosses (except for ecogeographic and phenological barriers, where such issues do not apply; Table 1). To obtain an overall value for barrier strengths involving F1 hybrid progeny, we averaged RI‐values from EL^♀^ × VU^♂^ and VU^♀^ × EL^♂^ hybrids (see for instance Kay 2006). Following Lowry et al. (2008), we quantified species‐ and morph‐dependent asymmetries, respectively, as the absolute values of the differences for the strength of a given barrier between reciprocal crosses and between L‐ and S‐morphs. The statistical significance of asymmetries was tested using Kruskal–Wallis tests or GLMMs with contrasts (Table 1; SPSS version 20.0.0; IBM Corp., Armonk, NY). In all GLMMs, we used random effects to account for hierarchical data structure, Satterthwaite's method to determine the approximate denominator degree of freedom for unbalanced data sets, and sequential Bonferroni correction to account for multiple tests (Table 1).

**Table 1:**
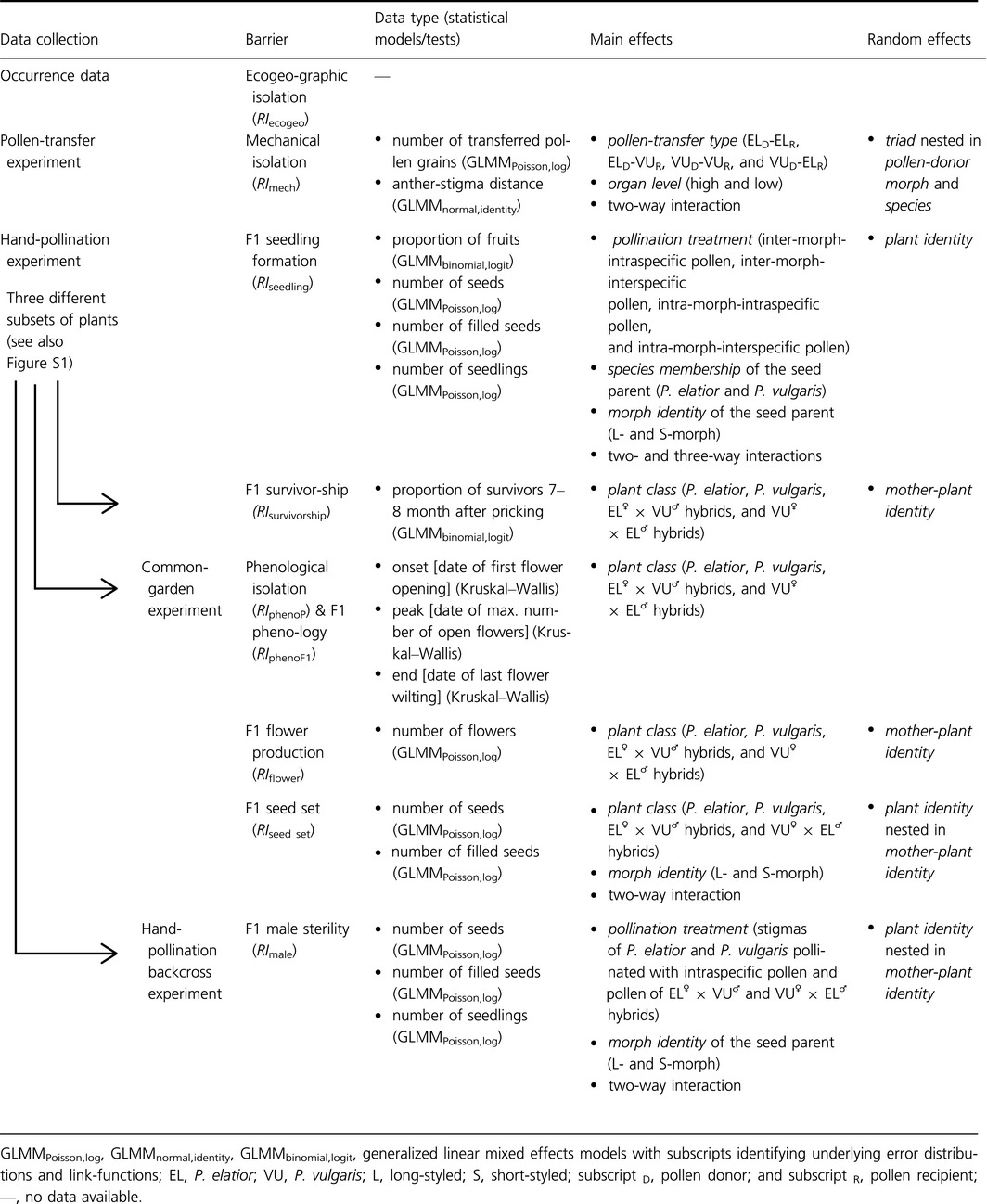
Data collection, experiments, and details of statistical analyses used to estimate reproductive barriers between *Primula elatior* and *P. vulgaris*. See also Figures S5–S9 and Tables S2–S3

#### Premating barriers

##### Ecogeographic isolation (RI_ecogeo_)

To estimate this reproductive barrier, we analyzed 23 578 occurrence records in 2907 1 × 1 km grid cells provided by the fine‐scale data base of the National Center for Information on the Swiss Flora (www.infoflora.ch). The 1 × 1 km grid cells represent a scale at which pollen of *P. vulgaris* and *P. elatior* is transported (max. distance: ~1.1 km and ~650 m, respectively; Van Geert et al. 2010; Van Rossum and Triest 2012). We adjusted the method of Ramsey et al. (2003) to fit grid‐based data and calculated RI_ecogeo_ with the equation 

(1) $${\text{RI}}_{\mathrm{ecogeo}}=1-\left(\frac{S}{S+U}\right)$$

of Sobel and Chen (2014), where *S* represents the number of grid cells that contain both species and *U* those that contain either *P. elatior* or *P. vulgaris*.

##### Phenological isolation (RI_phenoP_ and RI_phenoF1_)

A single common‐garden experiment was designed to estimate phenological isolation between *P. elatior* and *P. vulgaris* under sympatry (RI_phenoP_) and between parents and F1 hybrids (RI_phenoF1_); hence, these reproductive barriers are described together. The same experiment was also used to quantify relative hybrid fitness (*i.e.,* flower production and seed set: RI_flower_ and RI_seed set_, see below). For experimental plants, we used the offspring of the hand‐pollination experiment employed to quantify seed developmental isolation (see below and Table 1; for full experimental design of manual crosses, see Table S1). In November 2013, we transplanted 144 plants from Zürich to a locality in Niederönz (Switzerland). In spring 2014, 131 plants bloomed and produced a total of 2501 flowers. From 74 plants (20, 22, 17, and 15 plants of *P. elatior*,* P. vulgaris*, EL^♀^ × VU^♂^, and VU^♀^ × EL^♂^ hybrids, respectively; Table 1, Figure S1), we counted weekly the number of plants with open flowers and the number of open flowers per plant, for a total of 10 census days during the entire blooming period (28 February 2014–25 April 2014). First, we tested whether onset (*i.e.,* date of first flower opening), peak (*i.e.,* date of maximal number of open flowers), and end of flowering (*i.e.,* date of last flower wilting) differed between *P. elatior*,* P. vulgaris* and F1 hybrids (Table 1). Secondly, we quantified whether flowering asynchronies between parents restrict the formation of F1 hybrids (RI_phenoP_) and whether flowering asynchronies between F1 hybrids and parents restrict the formation of backcrosses (RI_phenoF1_). We assumed all flowers to be equally likely to mate, because individual flowers of parents and F1 hybrids remain open and receptive for at least 2 weeks (Eisikowitch and Woodell 1974; Taylor and Woodell 2008) and produce similar numbers of pollen grains (Ornduff 1979; Schou 1983; Piper and Charlesworth 1986). Assuming that parents and F1 hybrids occur in balanced morph ratios, we calculated how much *P. elatior* and *P. vulgaris* are phenologically isolated from each other (RI_phenoP_) and how much F1 hybrids are phenologically isolated from either parent (RI_pheno‐i_) and, *vice versa,* how much either parent is phenologically isolation from the F1 hybrids (RI_pheno‐ii_) with the equation 

(2) $${\text{RI}}_{\mathrm{pheno}}=1-2\sum_{i}\left(\frac{A_{i}}{A_{\mathrm{total}}}\times \frac{B_{i}}{A_{i}+B_{i}}\right)$$

of Sobel and Chen (2014) where (*A*_*i*_/*A*_ total_) refers to the proportion of open flowers of taxon *A* on day *i* in relation to their total abundance throughout the entire blooming period and (*B*_*i*_/*A*_*i*_ + *B*_*i*_) refers to the relative abundance of open flowers of taxon *B* on day *i*. Thus, RI_pheno‐i_ calculates the probability of gene flow within F1 hybrids (*F1 × F1*) and from F1 hybrids to either parent (*F1 × parent*), and RI_pheno‐ii_ calculates the probability of gene flow within each of the two parents (*parent* × *parent*) and from each of the two parents to the F1 hybrids (*parent* × *F1*). Using *F1 × parent* (*H*) and *parent* × *parent* (*C*), we estimated RI_phenoF1_ with the general equation to calculate reproductive isolation

(3) $$\text{RI}=1-2\times \left(\frac{H}{H+C}\right)$$

of Sobel and Chen (2014).

##### Mechanical isolation (RI_mech_)

To compare the intra‐ versus interspecific pollen movement between both low and high reciprocal organs, we performed a pollen transfer experiment in a walk‐in flight cage at the University of Zürich (Switzerland) in spring 2009. As pollen vector, we used the solitary bee *Anthophora plumipes* Pallas 1772 (Hymenoptera: Anthophoridae), because it frequently forages on both *Primula* species (Van Geert et al. 2010; Van Rossum et al. 2011) and can reach nectar at the bottom of the corolla tubes with its long tongue (Knuth 1909), effecting cross‐pollination between reciprocal morphs of heterostylous species (*e.g.,* Simón‐Porcar et al. 2014). All the numerous *Anthophora* bees and bumblebee queens, the other principal flower visitors, observed under natural and experimental conditions approach flowers by lowering proboscis and rostral part of the head into the corolla‐tube opening (B. Keller pers. obs.; Keller et al. 2014). Thus, all principal bee pollinators handle flowers in the same way.

We used 200 potted plants of each species obtained from a wild‐plant nursery (Vogt Stauden, Erlenbach). Flowers were kept in a pollinator‐free environment until the experiment. We used 35 male bees that were captured in the botanical garden. Experimental bees represent a random subsample of the naturally occurring bee population. Bees were kept in individual containers, cooled for ease of handling, and used multiple times, giving them time to groom and clean between triads (see below).

The quantification of mechanical isolation requires the precise measurement of pollen grains transferred between anthers and stigmas of two plant species by individual pollinators (*i.e.,* flower‐to‐flower pollen transfer; Campbell and Aldridge 2006). To achieve this goal, we used a set of three flowers (triad) comprising one pollen‐donor and two pollen‐recipient flowers of the reciprocal morph (one from the same species and one from the other species) as our basic experimental unit. The soundness of our experimental design depended on the ability to compare the number of pollen grains transferred to the two recipient flowers as precisely as possible. With free‐foraging insects, the length of flights or the intensity of grooming behavior cannot be controlled, as bees are more likely to groom while flying than while walking, and grooming reduces pollen carryover (Thomson et al. 1986). Therefore, we sacrificed the realism of flying bees in exchange for experimental feasibility and soundness by presenting flowers so that bees could walk from one flower to the next and excluded all trials in which bees flew and/or groomed between flowers. Our experimental design accounts for variation in number of deposited pollen grains dependent on pollinator visitation sequence (Lau and Bosque 2003) and provides the clearest picture of how the placement of floral organs affects pollen transfer (Campbell and Aldridge 2006).

A complete experiment consisted of eight triads divided into two experimental runs, one per species (Table 2; Figure S2). Triads were performed in random order and experiments were replicated ten times. We used intact pollen‐recipient flowers, for the removal of anthers might affect how deeply pollinators can probe flowers, influencing pollen transfer patterns. Size differences between pollen of L‐ and S‐flowers allowed us to discriminate intermorph pollen from self‐ and intramorph pollen (Figure S3). After executing each triad, flowers were dissected, the height of anther midpoints and stigma bases measured to quantify anthers–stigma (AS) distances between donor and recipient flowers, and stigma squashes prepared to count numbers of transferred pollen grains (see Keller et al. 2014).

**Table 2:** Mechanical isolation: Experimental design to compare the intra‐ and interspecific pollen transfer between both high and low reciprocal organs of *Primula elatior* and *Primula vulgaris* (see also Figure S2). Each experiment was replicated 10 times

Experimental run	Triad	Organ level	Species		
			Pollen‐donor flower	First pollen‐recipient flower	Second pollen‐recipient flower
EL‐run	I	Low	EL	VU	EL
	II			EL	VU
	III	High	EL	VU	EL
	IV			EL	VU
VU‐run	V	Low	VU	VU	EL
	VI			EL	VU
	VII	High	VU	VU	EL
	VIII			EL	VU

EL, *P. elatior*; VU, *P. vulgaris*; High, anthers of S‐morph flowers and reciprocal stigmas of L‐morph flowers; Low, anthers of L‐morph flowers and reciprocal stigmas of S‐morph flowers.

These data allowed us to test whether AS distances (absolute values) are larger (morphological prerequisite for mechanical isolation in heterostylous species; see Keller et al. 2012) and number of transferred pollen grains lower (quantification of the strength of mechanical isolation) between inter‐ than intraspecific reproductive organs (Table 1). We calculated RI_mech_ with equation (3), where *H* and *C* refer to number of pollen grains deposited on inter‐ and intraspecific stigmas, respectively. Finally, to compare the sexual organ reciprocity of experimental versus natural plants used in a previous study (Keller et al. 2012), we calculated intra‐ and interspecific reciprocity for both sets of plants following Richards and Koptur (1993; Table S1).

#### Postmating barriers

##### F1 seedling formation (i.e., seed developmental isolation: RI_seedling_)

To compare the success of intra‐ versus interspecific crosses, we performed hand‐pollination experiments in a greenhouse at the University of Zürich in spring 2012. Experimental plants were raised from seeds collected in natural, allopatric Swiss populations that are situated in the general area where distributional ranges of the two species overlap (Fig. 2): seeds of *P. elatior* were collected in Thun (BE) and seeds of *P. vulgaris* in Arogno (TI). Four pollination treatments were executed on emasculated L‐ and S‐flowers of both species: intermorph–interspecific, intermorph–intraspecific, intramorph–interspecific, and intramorph–intraspecific (details in Figure S4). Emasculations were performed in early anthetic flowers by removing the corolla with attached anthers. Effectiveness of emasculation was confirmed experimentally: only three of 74 emasculated, unpollinated flowers produced fruits, each with few seeds. Each pollination treatment was repeated up to three times per plant, for a total of 389 hand pollinations, divided between 16 L‐ and 29 S‐plants of *P. elatior* and 24 L‐ and 22 S‐plants of *P. vulgaris*. On each experimental day, newly harvested pollen from at least five flowers per morph and species was collected and applied on receptive stigmas. Wilted flowers were bagged to prevent seed loss. We counted the number of ripe fruits, total seeds, and filled seeds (*i.e.,* full‐sized, dark brown seeds; see Valentine 1947) produced by each hand‐pollinated flower (hereafter collectively termed “reproductive output”). After vernalization (4°C, 3 months), 1177 seeds from 15 L‐ and 24 S‐plants of *P. elatior* and 1143 seeds from 16 L‐ and 18 S‐plants of *P. vulgaris* were germinated in a growth chamber (Sanyo MLR 351H; Panosonic Corp., Kadoma, Osaka, Japan; conditions: 55% humidity, 12‐h dark at 10°C and 12‐h light [22,000 LUX] at 18°C). Seedlings were counted 11–20 weeks after sowing, pricked into individual pots, and raised to maturity. Subsets of these plants were used to quantify phenological isolation and all barriers listed below (Table 1, Figure S1).

We tested whether reproductive output differed between the four pollination treatments (Table 1). For intramorph pollinations, we expected reproductive output to be low, but significantly higher in inter‐ than intraspecific crosses, if intramorph incompatibility reaction is weakened in interspecific crosses. For intermorph pollinations, we expected reproductive output to be significantly lower in inter‐ versus intraspecific crosses, if reproductive barriers at this stage prevented formation of hybrid seedlings. We calculated RI_seedling_ from the number of seedlings (intermorph pollinations only) with equation (3), where *H* and *C* refer to the number of F1 hybrid and parental seedlings, respectively.

##### F1 survivorship (RI_survivorship_)

To compare the survivorship of F1 hybrids versus parents, we counted the number of viable plants 7–8 months after seedlings were pricked (80, 245, 66, and 98 seedlings of *P. elatior*,* P. vulgaris*, EL^♀^ × VU^♂^ hybrids, and VU^♀^ × EL^♂^ hybrids, respectively). We tested whether survival to maturity differed between F1 hybrids and parents (Table 1) and calculated RI_survivorship_ with equation (3), where *H* and *C* refer to the proportion of surviving F1 hybrids and parents, respectively.

##### F1 phenology (RI_phenoF1_)

See above.

##### F1 flower production (RI_flower_)

To compare the production of flowers between F1 hybrids and parents, we counted the number of flowers per plant directly after the last flower wilted (74 plants; see RI_phenoF1_ above). We tested whether number of flowers differed between F1 hybrids and parents (Table 1). We calculated RI_flower_ with equation (3), where *H* and *C* refer to the number F1 hybrid and parental flowers, respectively.

##### F1 seed set (RI_seed set_)

To compare the seed sets of F1 hybrids versus parents, we randomly bagged three open‐pollinated, wilted flowers per plant for 66 of the 74 plants used to calculate RI_phenoF1_ above (33 L‐plants: *P. elatior*, 6; *P. vulgaris*, 11; EL^♀^ × VU^♂^, 9; VU^♀^ × EL^♂^, 7; and 33 S‐plants: *P. elatior*, 13; *P. vulgaris*, 5; EL^♀^ × VU^♂^, 8; VU^♀^ × EL^♂^, 7; Figure S1). Fruits were collected and seeds counted as described under RI_seedling_. We tested whether reproductive output differed between F1 hybrids and parents (*i.e.,* the female component of hybrid sterility following Scopece et al. 2008; Table 1). We calculated RI_seed set_ from number of filled seeds with equation (3), where *H* and *C* refer to the number of F1 hybrid and parental seeds, respectively.

##### F1 male sterility (RI_male_)

To quantify the male component of hybrid sterility, we performed hand‐pollination experiments in a greenhouse at the University of Zürich in spring 2014. Following Scopece et al. (2008, 2013), we compared success of intraspecific versus backcross pollinations. Stigmas of *P. elatior* and *P. vulgaris* were pollinated with pollen of reciprocal flowers of *P. elatior*,* P. vulgaris*, EL^♀^ × VU^♂^ hybrids, and VU^♀^ × EL^♂^ hybrids, respectively (156 hand pollinations divided between 9 L‐ and 6 S‐plants of *P. elatior* and 19 L‐ and 18 S‐plants of *P. vulgaris*; Figure S1). On each experimental day, newly harvested pollen from up to three flowers was collected and applied on receptive stigmas. Fruits were collected, seeds counted, vernalized, and germinated as described under RI_seedling_. We tested whether reproductive output of parental plants differed when pollinated with F1 hybrid versus intraspecific pollen (Table 1). We calculated RI_male_ from number of seedlings with equation (3), where *H* and *C* refer to the number of backcross and parental seedlings, respectively.

#### Combined strength of pre‐ and postmating barriers

The combined strength of all premating barriers (RI_pre_), isolation under sympatry (RI_sympatry_), and total isolation (RI_tot_) were calculated with equation 

(4) $$\text{RI}=1-2\times \left(\frac{S\times H_{S}+U\times H_{U}}{S\times H_{S}+U\times H_{U}+S\times C_{S}+U\times C_{U}}\right)$$

of Sobel and Chen (2014), which considers *H* and *C* within shared (*H*_S_, *C*_S_) and unshared (*H*_U_, *C*_U_) space and/or time, respectively. The combined strength of all postmating barriers (RI_post_) was calculated with equation (3), where *H* and *C* refer to interspecific and intraspecific effects, respectively, each multiplied across all barrier types.

## Results

### Premating barriers

#### Ecogeographic isolation (RI_ecogeo_)

Ecogeographic isolation was stronger for *P. elatior* than for *P. vulgaris*, thus asymmetric between species (Tables 3, 4): 82.7% of the *P. elatior* grid cells did not contain *P. vulgaris*, while 59% of the *P. vulgaris* grid cells did not contain *P. elatior*. Morph‐dependent asymmetry was not tested, because it does not apply.

**Table 3:** Strengths of reproductive barriers between *Primula elatior* and *Primula vulgaris* for distribution (ecogeographic: RI_ecogeo_), flowering (phenology of parents: RI_phenoP_), pollen transfer (mechanical: RI_mech_), F1 seedling formation (seed developmental isolation: RI_seedling_), F1 survivorship (RI_*s*urvivorship_), and F1 reproduction, subdivided into phenology (RI_phenoF1_), flower production (RI_flower_), seed set (RI_seed set_), and male sterility (RI_male_). The combined strength of individual barriers is presented for all premating barriers (RI_pre_), all postmating barriers (RI_post_), all pre‐ and postmating barriers (RI_tot_), and all barriers occurring under sympatry (RI_sympatry_). RI*‐*values range from one (complete isolation: no interspecific gene flow) through zero (no isolation: equal probability of intra‐ and interspecific gene flow) to minus one (no isolation: all gene flow is interspecific; Sobel and Chen 2014)

	Stages in life cycle	Barrier name	*P. elatior*		*P. vulgaris*	
			L‐morph	S‐morph	L‐morph	S‐morph
Premating	Distribution	RI_ecogeo_	0.827		0.590	
	Flowering	RI_phenoP_	0.237		0.417	
	Pollen transfer	RI_mech_ a	0.100	−0.273	0.158	−0.136
Postmating	Formation of F1 hybrids	RI_seedling_ a	0.282	0.184	0.789	0.393
	Survivorship of F1 hybrids	RI_survivorship_ b	0.054		−0.097	
	Reproduction of F1 hybrids	RI_phenoF1_ b	−0.046		−0.136	
		RI_flower_ b	−0.125		−0.194	
		RI_seed set_ a ^,^ b	−0.116	0.092	0.223	0.317
		RI_male_ a ^,^ b	−0.088	−0.123	0.378	0.008
Total	Premating	RI_pre_ a	0.881	0.812	0.799	0.728
	Postmating	RI_post_ a	−0.034	0.036	0.851	0.311
	Pre‐ and postmating	RI_tot_ a	0.873	0.843	0.982	0.847
	Pre‐ and postmating without RI_ecogeo_	RI_sympatry_ a	0.283	0.065	0.949	0.587

L, long styled and S, short styled.

a RI was calculated separately for long‐ and short‐styled morphs, using the pollen‐receiving morph as reference.

b Averages across RI‐values of the two F1 hybrid classes (EL^♀^ × VU^♂^ and VU^♀^ × EL^♂^ hybrids; see Figures S5–S9).

**Table 4:** Strengths of morph‐ and species‐dependent asymmetries for the same reproductive barriers between *Primula elatior* and *Primula vulgaris* included in Table 3. Absolute values of the asymmetries were calculated following Lowry et al. (2008), with values <0.15 indicating symmetric barriers, values ≥0.15 indicating asymmetric barriers, and values >0.5 indicating highly asymmetric barriers (see Lowry et al. 2008)

	Stages in life cycle	Barrier name	Asymmetry between morphs		Asymmetry between species	
			*P. elatior*	*P. vulgaris*	L‐morph	S‐morph
Premating	Distribution	RI_ecogeo_	–		0.237	
	Flowering	RI_phenoP_	–		0.180	
	Pollen transfer	RI_mech_	0.373	0.294	0.058	0.137
Postmating	Formation of F1 hybrids	RI_seedling_	0.098	0.396	0.507	0.209
	Survivorship of F1 hybrids	RI_survivorship_	–		0.151	
	Reproduction of F1 hybrids	RI_phenoF1_	–		0.090	
		RI_flower_	–		0.069	
		RI_seed set_	0.208	0.094	0.339	0.225
		RI_male_	0.035	0.370	0.466	0.131
Total	Premating	RI_pre_	0.069	0.071	0.082	0.084
	Postmating	RI_post_	0.070	0.540	0.885	0.275
	Pre‐ and postmating	RI_tot_	0.030	0.135	0.109	0.004
	Pre‐ and postmating without RI_ecogeo_	RI_sympatry_	0.218	0.362	0.666	0.522

L, long styled; S, short styled; –, not applicable.

#### Phenological isolation of parents (RI_pheno_)

**Figure 3:**
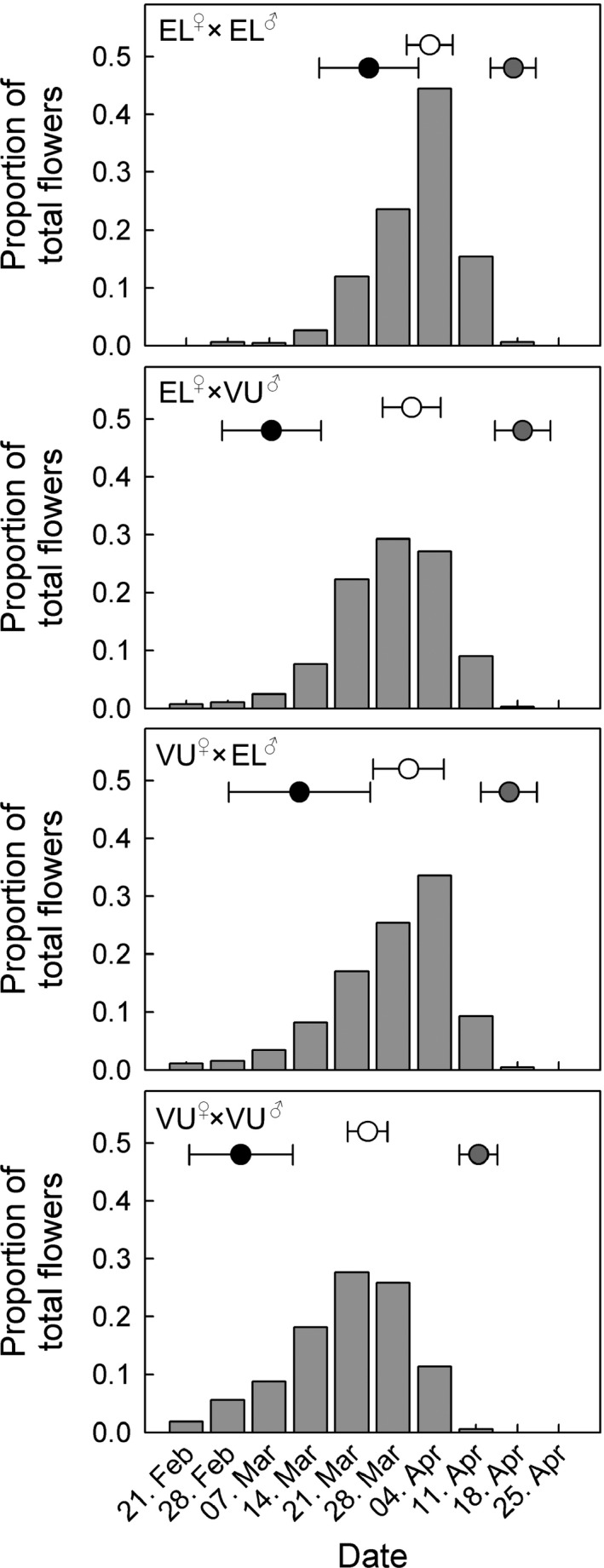
Parental and F1 phenology: Flowering phenology of *Primula elatior* (EL^♀^ × EL^♂^), *Primula vulgaris* (VU^♀^ × VU^♂^), and their F1 hybrids (EL^♀^ × VU^♂^ and VU^♀^ × EL^♂^) recorded weekly from plants in a common‐garden experiment, with means (circles) and standard deviations of onset (*i.e.,* date of first flower opening; black symbols), peak (*i.e.,* date of maximal number of open flowers; white symbols), and end (*i.e.,* date of last flower wilting; dark gray symbols) of flowering times. Percentages of total numbers of open flowers per census day (bars) are reported on the *y*‐axis for a total of 10 census days (*x*‐axis); 601 flowers in 20 plants of *P. elatior*, 852 flowers in 22 plants of *P. vulgaris*, 1287 flowers in 17 plants of EL^♀^ × VU^♂^ hybrids and 441 flowers in 15 plants of VU^♀^ × EL^♂^ hybrids were surveyed during their entire blooming period. Census data were used to calculate phenological isolation (RI_phenoP_) and F1 phenology (RI_phenoF1_; see text).

The flowering periods of *P. elatior* and *P. vulgaris* largely overlapped (Fig. 3). *Primula vulgaris* started, peaked, and ended blooming 22, 11, and 6 days, respectively, before *P. elatior* (Figs 3, S5). The blooming period of *P. vulgaris* was less nested within the one of *P. elatior* than *vice versa*. Consequently, RI_phenoP_ was stronger for the former than the latter, thus asymmetric between species (Tables 3, 4; morph‐dependent asymmetry not tested, because it does not apply).

#### Mechanical isolation (RI_mech_)

**Figure 4:**
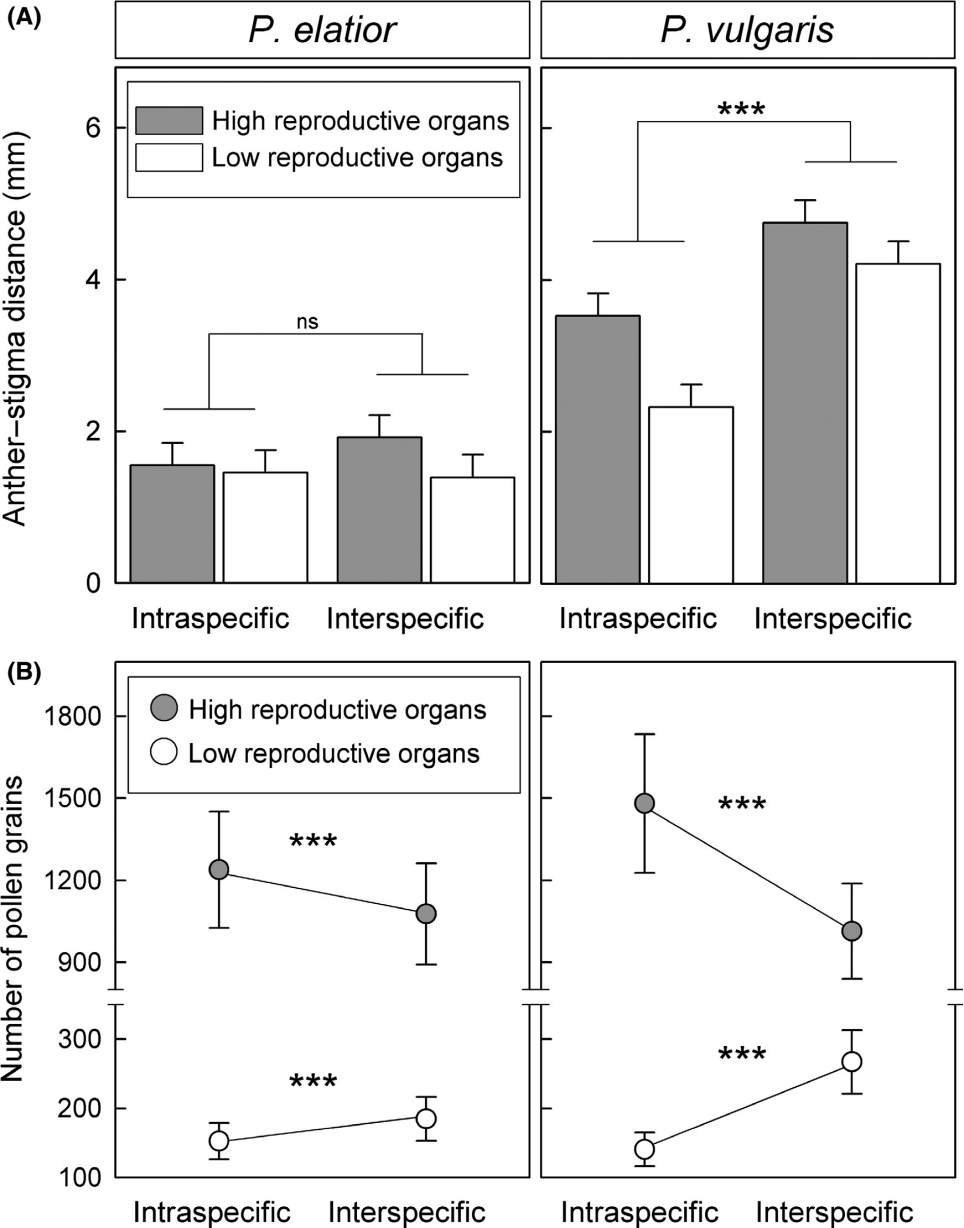
Pollen transfer and sexual organ distance: Mean values and standard errors (estimated from generalized linear mixed‐effects models) of (A) distances (absolute values) from anthers of pollen donors to stigmas of pollen recipients and (B) corresponding number of pollen grains transferred by *Anthophora plumipes* bees for both intra‐ and interspecific comparisons with *Primula elatior* (left panels) and *Primula vulgaris* (right panels) as pollen donors. Significance levels: *P *≤* *0.001 (***) or not significantly different *P *>* *0.05 (ns). Sequential Bonferroni correction was used to account for multiple tests. Mean values of number of pollen grains deposited on intra‐ and interspecific stigmas were used to calculate mechanical barriers (RI_mech_; see Table 3).

The intra‐ and interspecific reciprocity values of experimental plants were similar to those of plants from natural populations (Table S1). Anther–stigma distances between pollen donor and recipient flowers were larger between than within species, as expected; the differences were significant for *P. vulgaris*, but not significant for *P. elatior* (Fig. 4A; Table S1; for GLMM results see Table S2a). We counted a total of 133 612 pollen grains exported from anthers to reciprocal stigmas across all pollen transfer experiments (*P. elatior*: 64 526; *P. vulgaris*: 69 086). High anthers exported significantly fewer pollen grains to inter‐ than intraspecific reciprocal stigmas, as expected, while low anthers exported significantly more pollen grains to inter‐ than intraspecific reciprocal stigmas (Fig. 4B; for GLMM results, see Table S2b). In both species, RI_mech_ was thus positive for pollen recipients with high stigmas (L‐morph), but negative for recipients with low stigmas (S‐morph; Table 3). Mechanical isolation was therefore asymmetric between morphs, as predicted (see Introduction), but not between species (Table 4).

### Postmating barriers

#### F1 seedling formation (*i.e*., seed developmental isolation: RI_seedling_)

**Figure 5:**
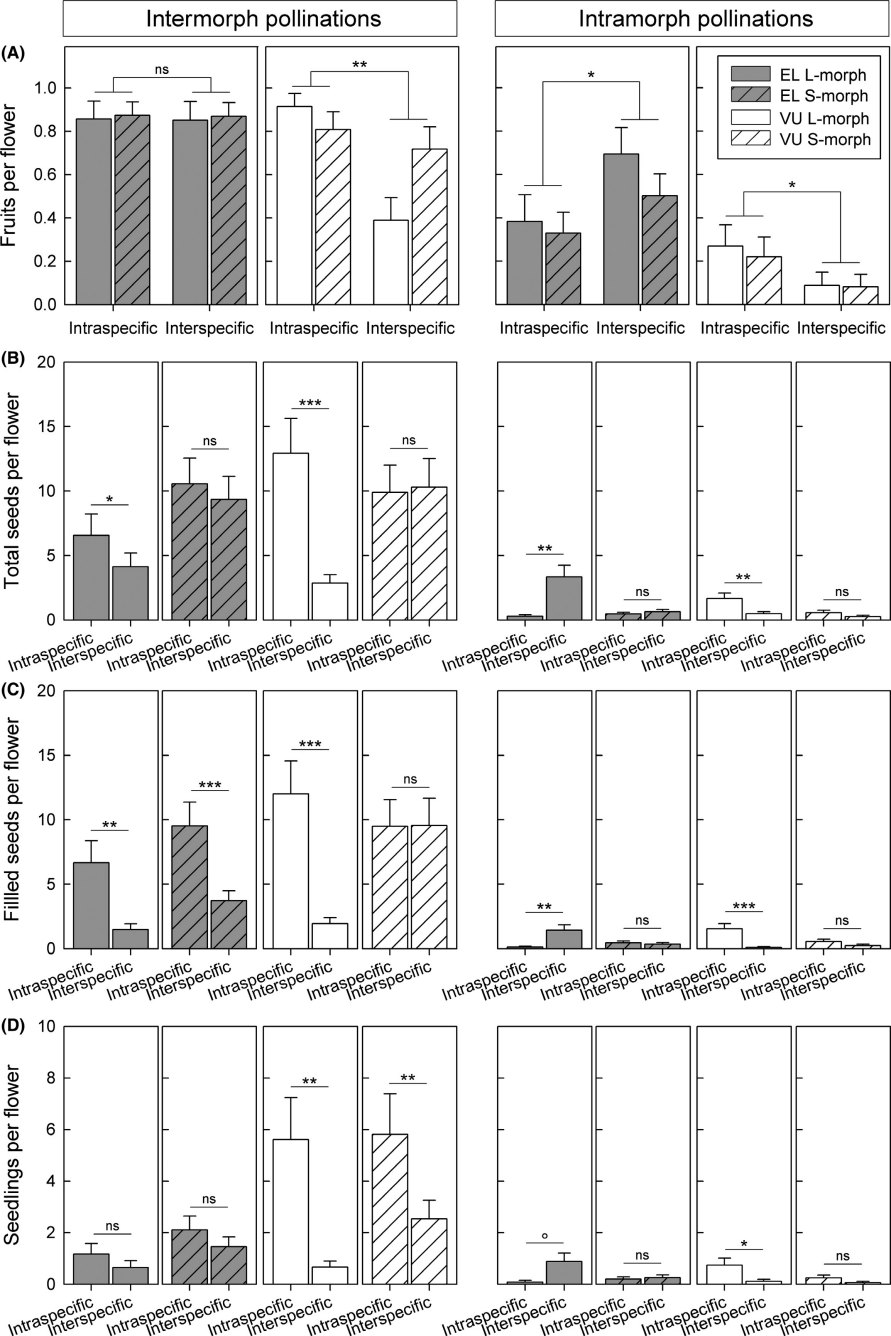
F1 seedling formation and intramorph incompatibility in intra‐ versus interspecific crosses: means and standard errors (estimated from generalized linear mixed‐effects models) of number of (A) fruits, (B) seeds, (C) filled seeds, and (D) seedlings produced per flower pollinated with intermorph (compatible; left panels) and intramorph (incompatible; right panels) pollen from intra‐ and interspecific crosses using *Primula elatior* and *Primula vulgaris* as pollen recipients, respectively. Traits (B–D), showing significant three‐way interaction (see Table S3), are presented in four panels each; trait (A), without significant three‐way interaction, in two panels. Significance levels: *P *≤* *0.001 (***), *P *≤* *0.01 (**), *P *≤* *0.05 (*), *P *≤* *0.08 (°), or not significantly different *P *>* *0.08 (ns). Sequential Bonferroni correction was implemented to account for multiple tests. Mean values of numbers of F1 hybrid and intraspecific seedlings from intermorph crosses were used to calculate reproductive isolation at this stage (RI_seedling_) and reproductive success of intramorph pollinations was used to assess whether intramorph incompatibility is maintained in interspecific crosses (see Table 3).

Reproductive success differed significantly among the four pollination treatments (Fig. 5; GLMM results in Table S3). For intramorph pollinations, reproductive success was generally low, as expected (Fig. 5 right panels); additionally, short‐styled morphs of *P. elatior* and both morphs of *P. vulgaris* had significantly lower reproductive success in inter‐ than intraspecific crosses and/or the difference was not significant, while L‐morphs of *P. elatior* had significantly higher reproductive success in inter‐ than intraspecific crosses, indicating that intramorph incompatibility is weakened in interspecific crosses of *P. elatior*. For intermorph pollinations, reproductive success was significantly lower in inter‐ than intraspecific crosses, as expected (Fig. 5, left panels), but in the following cases, differences between inter‐ and intraspecific crosses were not significant: numbers of fruits in both morphs of *P. elatior* (Fig. 5A), seeds in the S‐morph of both *P. elatior* and *P. vulgaris* (Fig. 5B), filled seeds in the S‐morph of *P. vulgaris* (Fig. 5C), and seedlings in both morphs of *P. elatior* (Fig. 5D). To summarize, fewer seedlings were formed in inter‐ than intraspecific crosses, and values of RI_seedling_ were higher in *P. vulgaris* than *P. elatior* and in L‐ than S‐morphs (Table 3), thus asymmetric between species (both morphs) and morphs (only for *P. vulgaris*; Table 4).

#### F1 survivorship (RI_survivorship_)

More plants of *P. elatior* and EL^♀^ × VU^♂^ hybrids survived than plants of *P. vulgaris* and VU^♀^ × EL^♂^ hybrids (GLMM results in Figure S6). The difference was significant between *P. elatior* and *P. vulgaris* and between *P. vulgaris* and EL^♀^ × VU^♂^ hybrids. Mean RI_survivorship_ favored introgression over isolation for *P. vulgaris*, while it was close to zero and positive for *P. elatior* (Table 3). Thus, RI_survivorship_ was asymmetric between species (Table 4; morph‐dependent asymmetry not tested).

#### F1 phenological isolation (RI_phenoF1_)

F1 hybrids started blooming with *P. vulgaris,* but peaked and ended blooming with *P. elatior* (Figs 3, S5); thus, mean RI_phenoF1_ favored introgression over isolation similarly in both species (Tables 3, 4; morph‐dependent asymmetry not tested, because it does not apply).

#### F1 flower production (RI_flower_)

EL^♀^ × VU^♂^ hybrids had significantly more flowers than their parents, while the number of flowers did not differ between VU^♀^ × EL^♂^ hybrids and parents (GLMM results in Figure S7). Thus, mean RI_flower_ favored introgression over isolation similarly in both species (Tables 3, 4; morph‐dependent asymmetry not tested).

#### F1 seed set (RI_seed set_)

The number of total seeds and filled seeds did not differ between F1 hybrids and *P. elatior*, but F1 hybrids had significantly fewer total seeds (VU^♀^ × EL^♂^ hybrids only) and filled seeds (both hybrids) than *P. vulgaris* (GLMM results in Figure S8). Mean RI_seed set_ reduced gene flow to *P. vulgaris*, while it was weak or negative in *P. elatior* (Table 3). Thus, RI_seed set_ was asymmetric between species (both morphs) and morphs (*P. elatior* only; Table 4). The latter, however, was not statistically supported (morph effects were not significant in the GLMM; Figure S8).

#### F1 male sterility (RI_male_)

Reproductive output of both *P. elatior* and *P. vulgaris* did not differ significantly between flowers that were pollinated with F1 hybrid or intraspecific pollen (GLMM results in Figure S9), with the following exceptions: L‐flowers of *P. vulgaris* produced significantly fewer seeds and filled seeds when pollinated with EL^♀^ × VU^♂^ hybrid than with intraspecific pollen (Figure S9A,B) and significantly fewer seedlings when pollinated with both EL^♀^ × VU^♂^ and VU^♀^ × EL^♂^ hybrid than with intraspecific pollen (Figure S9C). Mean RI_male_ reduced gene flow by 37% in L‐morphs of *P. vulgaris,* while it was weak in S‐morphs of the same species and favored introgression over isolation in *P. elatior* (Table 3). Thus, RI_male_ was asymmetric between species (L‐morph only) and morphs (*P. vulgaris* only; Table 4).

### Combined strength of pre‐ and postmating barriers

Total isolation between *P. elatior* and *P. vulgaris* is strong, but incomplete for both species (Table 3). Total isolation of *P. elatior* mainly depends on RI_ecogeo_, which reduces gene flow by 83%. Total isolation of *P. vulgaris* mainly depends on the combined effects of RI_ecogeo_, RI_phenoP_, RI_seedling_, RI_seed set_, and RI_male_ (L‐morph only), each reducing gene flow between 22% and 59% (Table 3). The five barriers are thus all asymmetric between species (Table 4). Barrier strengths also varied between morphs (Table 4). Statistically supported morph‐dependent asymmetries occurred in RI_mech_ for both species and in RI_seedling_ and RI_male_ for *P. vulgaris* (Figs 4B, 5D, S9C; Tables S2, S3D), with all three barriers being stronger in L‐ than S‐morphs (Table 3). Premating isolation is globally stronger than postmating isolation (RI_pre_ > RI_post)_ in both morphs of *P. elatior* and in the S‐morph of *P. vulgaris*, but not in the L‐morph of the latter species. Furthermore, RI is largely maintained under sympatry for *P. vulgaris*, but not for *P. elatior*; hence, RI_sympatry_ is asymmetric between species (Tables 3, 4). As RI_sympatry_ is also stronger in the L‐ than the S‐morph, gene flow between species is likely asymmetric between both species and morphs. Consequently, the strength of RI and relative importance of barriers to gene flow differ across the stages of the life cycle, between species, and between morphs.

## Discussion

We tested whether traits of heterostyly alter interspecific boundaries in distinct, possibly asymmetric ways. Specifically, we investigated three premating and six postmating barriers to gene flow, including mechanical barriers to pollen flow and postmating barriers involving F1 hybrids. To quantify mechanical isolation, we performed an experiment that provides the clearest picture to date of how the placement of sexual floral organs in heterostylous species affects pollen transfer. As pollen vector, we used *A. plumipes*, a common flower visitor of both species representing the long‐tongued bee pollinator type. Although it would have been ideal to use all known pollinator species and a more natural setup in the experiment aimed at comparing intra‐ versus interspecific pollen transfer between high and low sexual floral organs, the necessity of precise pollen counts precluded it. Nevertheless, our results represent the first contribution toward understanding whether the spatial separation of reproductive organs in heterostylous flowers can mechanically limit interspecific pollen movement between insect‐pollinated species with loose pollen grains. Importantly, differences in number of pollen grains transferred between the two sexual organ levels occur both between (Fig. 4) and within species (Brys and Jacquemyn 2015; Keller et al. 2014 and references therein), corroborating the soundness of our experimental approach and results.

Phenological, F1 flower production, and F1 seed‐set barriers were quantified in a common‐garden experiment. Flowering periods (Fig. 3) and numbers of flowers and seeds per individual were similar between the plants used in our experiment and those in natural populations (*e.g.,* Jacquemyn et al. 2002, 2009; Brys et al. 2007; Lauber and Wagner 2007; Taylor and Woodell 2008; Baeten et al. 2015), indicating that the common garden provided suitable habitat conditions for both investigated species. Additionally, strength and asymmetries of isolation under sympatry and postmating isolation are corroborated by previous studies between *P. elatior* and *P. vulgaris* from Britain (Valentine 1947, 1948), suggesting that estimates of reproductive barriers in our study are representative for the species. Nevertheless, further studies are needed to assess strength and variation of individual barriers across the entire species ranges.

Total reproductive isolation between the distylous *P. elatior* and *P. vulgaris* is high (Table 3), but less complete than what is generally observed in plants (Schemske 2010), corroborating previous reports on the frequent occurrence of hybrids and gene flow between the studied species (Gurney et al. 2007; Taylor and Woodell 2008; Schmidt‐Lebuhn et al. 2012). We document for the first time that, in addition to widely occurring species‐dependent asymmetries (Wirtz 1999; Tiffin et al. 2001; Turelli and Moyle 2007; Lowry et al. 2008), morph‐dependent asymmetries affect RI, especially under sympatry (Tables 3, 4). Below, we explain the contributions of individual barriers to shaping species boundaries, focusing on morph‐ and species‐dependent asymmetries, the unique role of heterostyly in reproductive isolation, and the implications of our findings for conservation and evolution in a changing world.

### Pre‐ and postmating contributions to reproductive isolation

It is well‐established that multiple reproductive barriers promote species divergence and maintenance (Coyne and Orr 2004), but the relative contributions of multiple pre‐ and postmating mechanisms to total isolation remain poorly understood and are unknown for heterostylous taxa. Premating barriers are expected to be stronger than postmating ones, because the former act earlier in the life cycle (Coyne and Orr 2004; Lowry et al. 2008; Baack et al. 2015). Indeed, our results support the general prediction of higher premating isolation in three of four cases, for RI_pre_ is stronger in both morphs of *P. elatior* and in the S‐morph of *P. vulgaris*, while RI_post_ prevails in the L‐morph of *P. vulgaris* (Table 3).

Premating barriers restrict opportunities for gamete encounters between species. Between *P. elatior* and *P. vulgaris*, ecogeographic isolation represents the premating mechanism of largest effect (Table 3), corroborating both theoretical expectations (Sobel and Chen 2014) and previous findings in other species (*e.g.,* Kay 2006; Sambatti et al. 2012; Sánchez‐Guillén et al. 2012; Sobel and Streisfeld 2015). Our results also confirm that small differences in the timing of flowering (Fig. 3) can decrease gene flow via restricting the temporal window available for interspecific pollinations (Carrió and Güemes 2014; Melo et al. 2014). The low strength of mechanical isolation detected between our study species (Table 3) also supports the results of previous studies that found this barrier to be especially weak in insect‐pollinated species with loose pollen (including primroses), suggesting that such species may be unable to achieve the high precision of pollen transfer required to effect strong mechanical barriers (Armbruster et al. 2009, 2014).

After interspecific pollen is transferred, fertilization may fail or hybrid seeds may not develop into seedlings (*e.g.,* Eaton 1973; Johnston et al. 1980; Lester and Kang 1998). Corroborating general findings (Marshall and Folsom 1991) and earlier results for *Primula* (*e.g.,* De Vries 1919/20; Valentine 1947), we discovered that incompatibilities in seed development represent pronounced isolating mechanisms (Fig. 5). After hybrid formation, hybrids may be unfit, meiosis may fail and/or backcrossed seeds may not develop into seedlings (Coyne and Orr 2004; Baack et al. 2015). Confirming earlier results for *P. elatior* and *P. vulgaris* in Britain (Valentine 1947), barriers after the formation of hybrids are weak or even favor introgression over isolation, especially for *P. elatior* (Table 3). Thus, species integrity rests primarily on barriers preventing hybrid formation in both species, but more conspicuously in *P. elatior*, while postmating barriers play a comparatively more important role in *P. vulgaris*. The relative contributions of pre‐ versus postmating barriers to reproductive isolation are thus species‐specific (*i.e.,* asymmetric).

### Morph‐dependent asymmetries, species‐dependent asymmetries, and how heterostyly contributes to reproductive isolation

The strength of reproductive barriers may be influenced by which species and morph serve as male or female parent. While species‐dependent asymmetries have been described in both animals and plants (Wirtz 1999; Tiffin et al. 2001; Turelli and Moyle 2007; Lowry et al. 2008), morph‐dependent asymmetries remain undocumented, likely because they can only be detected in hermaphroditic species with stable heteromorphism, such as heterostylous primroses.

We document species‐dependent asymmetries in RI_ecogeo_ (stronger for *P. elatior* than *P. vulgaris*) and in RI_phenoP_, RI_seedling_, RI_seed set_, and RI_male_ (all stronger for *P. vulgaris* than *P. elatior*; Tables 3, 4). Directionality in the formation of hybrid seeds or seedlings had been previously reported for monomorphic species (*e.g.,* Ramsey et al. 2003) and heteromorphic primroses (De Vries 1919/20; Valentine 1947; Eaton 1973; Ma et al. 2014; but see Heslop‐Harrison 1931). Corroborating the mentioned studies on primroses, we find the development of hybrid seeds and seedlings to be more hampered for *P. vulgaris* than for *P. elatior* (Fig. 5). Genomic imbalances causing asynchronous development of embryo and endosperm in one cross‐direction more than in the other likely explain species‐dependent asymmetries of hybrid seed formation in *Primula* (Valentine 1947). Finally, reproductive isolation under sympatry is considerably stronger for *P. vulgaris* (where RI_tot_ depends on the combined effects of several barriers) than for *P. elatior* (where RI_tot_ mainly depends on RI_ecogeo_; Table 3), possibly favoring asymmetric introgression between species (*e.g.,* Arnold et al. 2010).

Morph‐dependent asymmetries can only exist in sexually heteromorphic, hermaphroditic species, such as heterostylous primroses. Mechanical isolation between *P. vulgaris* and *P. elatior* is clearly affected by morph‐dependent asymmetries, for lower sexual organ reciprocity between species decreases interspecific pollen transfer for L‐morphs, as expected (see Introduction), while favoring introgression over isolation for S‐morphs (Fig. 4, Table 3). Indeed, the higher level of mechanical isolation for L‐flowers of both species might represent a consequence of higher selection to limit opportunities for access of interspecific pollen to ovules of flowers with exposed stigmas, which receive more pollen than flowers with sunken stigmas (Fig. 1A) both between (Fig. 4B) and within species of *Primula* (Keller et al. 2014). Similarly, decreased seed production in the L‐morphs of forest populations of *P. veris*, closely related to our study species, was explained in terms of decreased efficiency of pollen transfer to their exposed stigmas, possibly resulting from lower levels of sexual organ reciprocity in those populations (Brys and Jacquemyn 2015). Morph‐dependent asymmetries occur also postmatingly in seed developmental and male sterility barriers (Fig. 5, left panels; Table 4). The fact that such morph‐dependent effects, both pre‐ and postmating, were detected in both *P. elatior* and *P. vulgaris* raises the possibility that they might be directly or indirectly linked to the S‐locus. The recently published genetic map of the S‐locus in *P. vulgaris* (Li et al. 2015) and the draft genome of the phylogenetically close *Primula veris* (Nowak et al. 2015) provide crucial genomic resources to explore this notion.

The defining morphological and physiological traits of distyly are reciprocal herkogamy and intramorph incompatibility, respectively (Barrett 2002). Our results demonstrate, for the first time, that the morphological traits unique to heterostyly might impose limited mechanical isolation on one floral morph (namely, the L‐morph), while favoring interspecific pollen flow through the other (namely, the S‐morph; Table 3). Hence, the intensity of intermorph pollen transfer across species boundaries likely depends on the morph composition of populations coming into contact. In addition, inter‐ versus intraspecific comparisons of reproductive success from intramorph pollinations (Fig. 4, right panels) suggest that intramorph incompatibility persists across species boundaries, as expected (Chen 1999; De Nettancourt 2001; Ma et al. 2014), but appears to be weakened in L‐morph pollen recipients of *P. elatior*, opening up a possible backdoor to gene flow through intramorph pollen transfer across species boundaries. Both the morphological and physiological aspects of distyly may thus affect permeability of species boundaries in unique, complex ways.

### Asymmetries of reproductive barriers and pre‐ versus postmating mechanisms in changing environments

Ecogeographic isolation, the main reproductive barrier for many species (Schemske 2010), may break down when habitats are disturbed and/or species ranges change (Rhymer and Simberloff 1996; Abbott et al. 2013). Despite their ecological differences, populations of *P. vulgaris* and *P. elatior* often occur in close proximity (B. Keller, pers. obs.) and habitat disturbances might increase the probability that they come into contact, decreasing their currently high levels of ecogeographic isolation (Table 3). Hence, the strength and direction of RI under sympatry is crucial for species integrity.

Reproductive isolation in sympatry is asymmetric between both species (stronger in *P. vulgaris* than in *P. elatior*) and morphs (stronger in L‐ than S‐morphs; Tables 3, 4). These asymmetries may impact species boundaries especially when a small population of one species comes into contact with a large population of another species. Indeed, the former risks pollen swamping and high rates of introgression, becoming threatened by local extinction through hybridization, especially if its level of reproductive isolation from the latter is insufficient (Levin et al. 1996; Prentis et al. 2007; Balao et al. 2015). Extinction risk is thus particularly high in small heterostylous populations with unbalanced morph ratios. For example, small populations of *P. elatior* dominated by S‐plants co‐occurring with large populations of *P. vulgaris* may risk pollen swamping and severe introgression, because intraspecific pollen of L‐flowers is rare, interspecific pollen of L‐flowers is abundant (Fig. 3A), and postmating isolation is weak (Table 3). Human‐mediated habitat fragmentation progressively reduces population sizes of plant species worldwide (*e.g.,* Aguilar et al. 2006). Hence, the number of small populations is likely to continue to increase, potentially skewing morph ratios in heterostylous populations (*e.g.,* Jacquemyn et al. 2002; Meeus et al. 2012) and affecting the permeability of species boundaries.

Under sympatry, barriers might be especially susceptible to habitat alteration (Lamont et al. 2003; Franks and Weis 2009), for example, if they depend on blooming periods and plant–pollinator interactions that may vary in space and time (*e.g.,* Martin and Willis 2007; Marques et al. 2012; Natalis and Wesselingh 2013). As premating barriers often consist in specific habitat adaptations, they are thought to be more susceptible to environmental changes than intrinsic postmating barriers based on genetic incompatibilities (Turelli et al. 2001; Coyne and Orr 2004; Seehausen et al. 2014). Considering the sensitivity of premating barriers to environmental variation through time and space, genetically based postmating barriers (for example, seed developmental isolation between *P. elatior* and *P. vulgaris*; Fig. 4; Table 3) may be crucial to the maintenance of species boundaries over time (Widmer et al. 2008).

To conclude, the strength, yet lability of premating barriers in *P. elatior* and *P. vulgaris* are congruent with conceptual models of diversification suggesting that, while initiation of species divergence may be common, most newly formed lineages perish (Rosenblum et al. 2012). In the short term, traits linked with premating barriers may evolve readily and enable populations to diverge rapidly even in sympatry (*e.g.,* Savolainen et al. 2006). In the longer term, however, the susceptibility of premating barriers to changing environmental conditions makes it improbable that they alone can maintain a species' genetic integrity. Therefore, intrinsic postmating barriers are necessary to ensure species survival over broader temporal and geographic scales, although even they can fluctuate across species ranges (Widmer et al. 2008; Cutter 2012). The varying strength of both pre‐ and postmating barriers through time and space is consistent with the idea that much species divergence may be ephemeral (Rosenblum et al. 2012), contributing little to long‐term evolutionary patterns.

## Data accessibility

The raw data of all experiments are deposited in the Dryad Digital Repository: http://dx.doi.org/10.5061/dryad.c715s.

## Conflict of Interest

None declared.

## Supporting information


**Table S1.** Intra – and interspecific sexual organ reciprocity of experimental plants and plants from natural Swiss populations of *P. elatior* and *P. vulgaris*.
**Table S2.** Results of generalized linear mixed‐effects models testing whether anther–stigma distance and pollen transfer differ between *pollen transfer types* and *organ level*s.
**Table S3.** Results of generalized linear mixed‐effects models testing whether reproductive output differs between *species*,* morphs*, and *pollination treatments*.
**Figure S1.** Experimental design for manual crosses used to estimate reproductive barriers between *P. elatior* and *P. vulgaris*.
**Figure S2.** Experimental design used to compare the intra‐ and interspecific pollen transfer between *P. elatior* and *P. vulgaris*.
**Figure S3.** Distribution of pollen grain sizes of L‐ and S‐ flowers of *P. elatior* and *P. vulgaris*.
**Figure S4.** Graphical representation of the four pollination treatments used to estimate F1 seedling formation and interspecific intra‐morph incompatibility.
**Figure S5.** Onset, peak, and end of the flowering period of *P. elatior*,* P. vulgaris*, and F1 hybrids and results of statistical tests assessing phenological differences between parents and hybrid offspring.
**Figures S6–S9.** Relative fitness of *P. elatior*,* P. vulgaris*, and F1 hybrids and results of statistical tests assessing differences between parents and hybrid offspring: F1 survivorship (Figure S6), F1 flower production (Figure S7), F1 seed set (Figure S8), and F1 male sterility (Figure S9).
