# Supplementary material for: Both morph‐ and species‐dependent asymmetries affect reproductive barriers between heterostylous species
Source: Ecol Evol. 2016 Aug 4;6(17):6223–44. doi: 10.1002/ece3.2293 (PMC5016645; doi:10.1002/ece3.2293)
Supplement: Supplementary file 1 — Table S1. Intra – and interspecific sexual organ reciprocity of experimental plants and plants from natural Swiss populations of P. elatior and P. vulgaris. Table S2. Results of generalized linear mixed‐effects models testing whether anther–stigma distance and pollen transfer differ between pollen transfer types and organ levels. Table S3. Results of generalized linear mixed‐effects models testing whether reproductive output differs between species, morphs, and pollination treatments. Figure S1. Experimental design for manual crosses used to estimate reproductive barriers between P. elatior and P. vulgaris. Figure S2. Experimental design used to compare the intra‐ and interspecific pollen transfer between P. elatior and P. vulgaris. Figure S3. Distribution of pollen grain sizes of L‐ and S‐ flowers of P. elatior and P. vulgaris. Figure S4. Graphical representation of the four pollination treatments used to estimate F1 seedling formation and interspecific intra‐morph incompatibility. Figure S5. Onset, peak, and end of the flowering period of P. elatior, P. vulgaris, and F1 hybrids and results of statistical tests assessing phenological differences between parents and hybrid offspring. Figures S6–S9. Relative fitness of P. elatior, P. vulgaris, and F1 hybrids and results of statistical tests assessing differences between parents and hybrid offspring: F1 survivorship (Figure S6), F1 flower production (Figure S7), F1 seed set (Figure S8), and F1 male sterility (Figure S9). [file ECE3-6-6223-s001.docx]

**Supporting information**

**Tables**

**Table S1.** Mechanical isolation: Intra- and interspecific reciprocity between sexual organs in *Primula elatior* (EL) and *P. vulgaris* (VU) calculated with the index of Richards and Koptur (1993) for high (H) and low (L) reproductive organs of experimental plants and plants from natural Swiss populations (data from Keller et al. 2012). As stigmas for pollen quantification were harvested in the experiment (*i.e.,* stigma height is measured between base of gynoecium and stigma base), mean values of natural plants were re-calculated for middles of anthers (A), and tips and bases of stigmas (S) from raw data of Keller et al. (2012) using 132 and 123 long- and 120 and 123 short-styled flowers of *P. elatior* and *P. vulgaris*, respectively. Reciprocity was calculated from experimental- and species-wide means of floral measurements with the equations *R*_H_ = (A_H_ – S_H_)/(A_H_ + S_H_) for high reproductive organs and *R*_L_ = (A_L_ – S_L_)/(A_L_ + S_L_) for low reproductive organs.

|  | Organ level | Natural plants | | Experimental plants |
| --- | --- | --- | --- | --- |
|  |  | Stigma tip | Stigma base | Stigma base |
| EL-EL | High | 0.020 | 0.050 | 0.020 |
|  | Low | 0.049 | 0.096 | 0.108 |
| VU-VU | High | 0.030 | 0.063 | 0.095 |
|  | Low | 0.032 | 0.078 | 0.105 |
| EL-VU | High | -0.095 | -0.062 | -0.054 |
|  | Low | -0.131 | -0.084 | -0.043 |
| VU-EL | High | 0.144 | 0.173 | 0.168 |
|  | Low | 0.209 | 0.254 | 0.251 |
| Index varies between –1 and 1; perfect reciprocity = 0; positive values indicate that the mean anther position is higher than the mean stigma position, while negative values indicate the opposite. In interspecific comparisons (*e.g.,* EL-VU), anthers of the species mentioned first are compared to stigmas of the second species. | | | | |

**Table S2.** Mechanical isolation: Results of generalized linear mixed effects models testing whether (A) anther-stigma distance (absolute values) and (B) pollen transfer differ between flowers of the same *vs.* different species for pollen donors of *Primula elatior* and *P. vulgaris* (*pollen-transfer type*) and high *vs.* low organs (*organ level*).

|  | Source | d.f. | *F* | *P* |
| --- | --- | --- | --- | --- |
| (A) | Pollen-transfer type (P) | 3,152 | 39.591 | ≤0.001 |
|  | Organ level (O) | 1,152 | 5.875 | 0.017 |
|  | P×O | 3,152 | 1.539 | 0.207 |
| (B) | Pollen-transfer type (P) | 3,152 | 48.953 | ≤0.001 |
|  | Organ level (O) | 1,152 | 120.443 | ≤0.001 |
|  | P×O | 3,152 | 777.964 | ≤0.001 |

**Table S3.** F1 seedling formation and interspecific intramorph incompatibility: Results of generalized linear mixed effects models testing whether formation of (A) fruits, (B) total seeds, (C) filled seeds, and (D) seedlings differ between *Primula elatior* and *P. vulgaris* (*species membership of seed parent*), L- and S-morphs (*morph identity of seed parent*) and the following four *pollination treatments:* intermorph intraspecific pollination, intermorph interspecific pollination, intramorph intraspecific pollination, and intramorph interspecific pollination.

|  | Source | d.f. | *F* | *P* |
| --- | --- | --- | --- | --- |
| (A) | Species membership of seed parent (S) | 1,107 | 14.346 | ≤0.001 |
|  | Morph identity of seed parent (M) | 1,107 | 0.058 | 0.810 |
|  | Pollination treatment (P) | 3,353 | 24.351 | ≤0.001 |
|  | S × M | 1,107 | 0.112 | 0.739 |
|  | S × P | 3,353 | 4.306 | 0.005 |
|  | M × P | 3,353 | 1.091 | 0.353 |
|  | S × M × P | 3 353 | 0.784 | 0.524 |
| (B) | Species membership of seed parent (S) | 1,96 | 0.083 | 0.774 |
|  | Morph identity of seed parent (M) | 1,96 | 0.091 | 0.764 |
|  | Pollination treatment (P) | 3,353 | 277.420 | ≤0.001 |
|  | S × M | 1,96 | 0.218 | 0.642 |
|  | S × P | 3,353 | 23.268 | ≤0.001 |
|  | M × P | 3,353 | 44.821 | ≤0.001 |
|  | S × M × P | 3,353 | 16.424 | ≤0.001 |
| (C) | Species membership of seed parent (S) | 1,121 | 0.577 | 0.449 |
|  | Morph identity of seed parent (M) | 1,121 | 1.425 | 0.235 |
|  | Pollination treatment (P) | 3,353 | 256.601 | <0.001 |
|  | S × M | 1,121 | 0.001 | 0.979 |
|  | S × P | 3,353 | 12.967 | <0.001 |
|  | M × P | 3,353 | 28.334 | <0.001 |
|  | S × M × P | 3,353 | 14.558 | <0.001 |
| (D) | Species membership of seed parent (S) | 1,103 | 0.693 | 0.407 |
|  | Morph identity of seed parent (M) | 1,103 | 0.063 | 0.803 |
|  | Pollination treatment (P) | 3,297 | 80.860 | <0.001 |
|  | S × M | 1,103 | 0.263 | 0.609 |
|  | S × P | 3,297 | 15.533 | <0.001 |
|  | M × P | 3,297 | 6.812 | <0.001 |
|  | S × M × P | 3,297 | 3.268 | 0.022 |

**Figures**


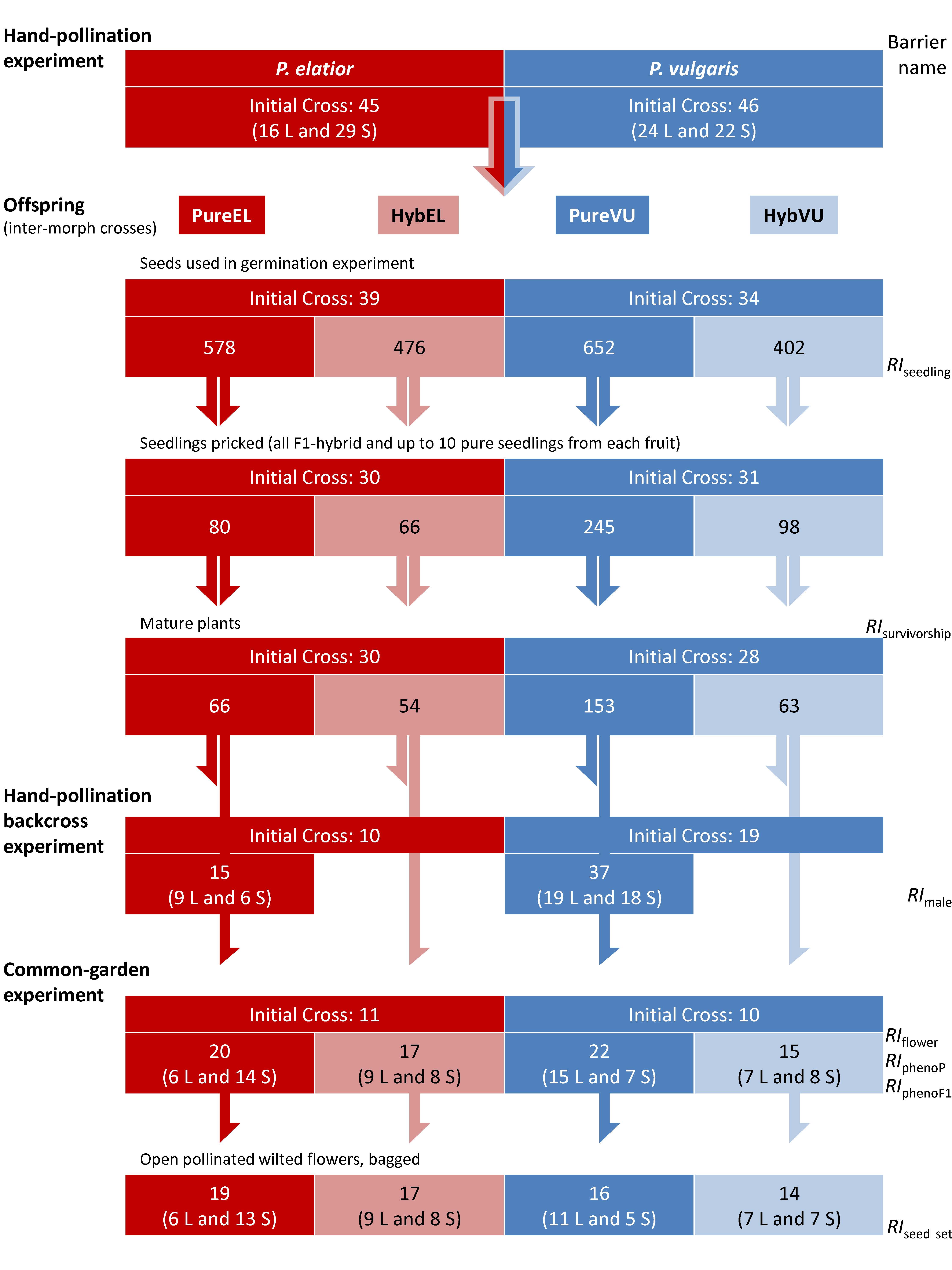


**Figure S1.** Experimental design for manual crosses used to estimate reproductive barriers: number of plants used in hand-pollination experiments to quantify strength of F1 seed formation (*RI*_seedling_) between *Primula elatior* and *P. vulgaris* (initial cross) and number of plants with their provenance (plants used in the initial crosses) used in subsequent experiments (offspring) to quantify F1 survivorship (*RI*_survivor_), F1 male sterility (*RI*_male_), F1 flower production (*RI*_flower_), phenological isolation under sympatry (*RI*_phenoP_), F1 phenology (*RI*_phenoF1_), and F1 seed set (*RI*_seed set_). Abbreviations: S = short-styled, L = long-styled, PureEL = offspring of the initial EL^♀^×EL^♂^ cross, pureVU = offspring of the initial VU^♀^×VU^♂^ cross, HybEL = offspring of the initial EL^♀^×VU^♂^ cross, and HybVU = offspring of the initial VU^♀^×EL^♂^ cross.


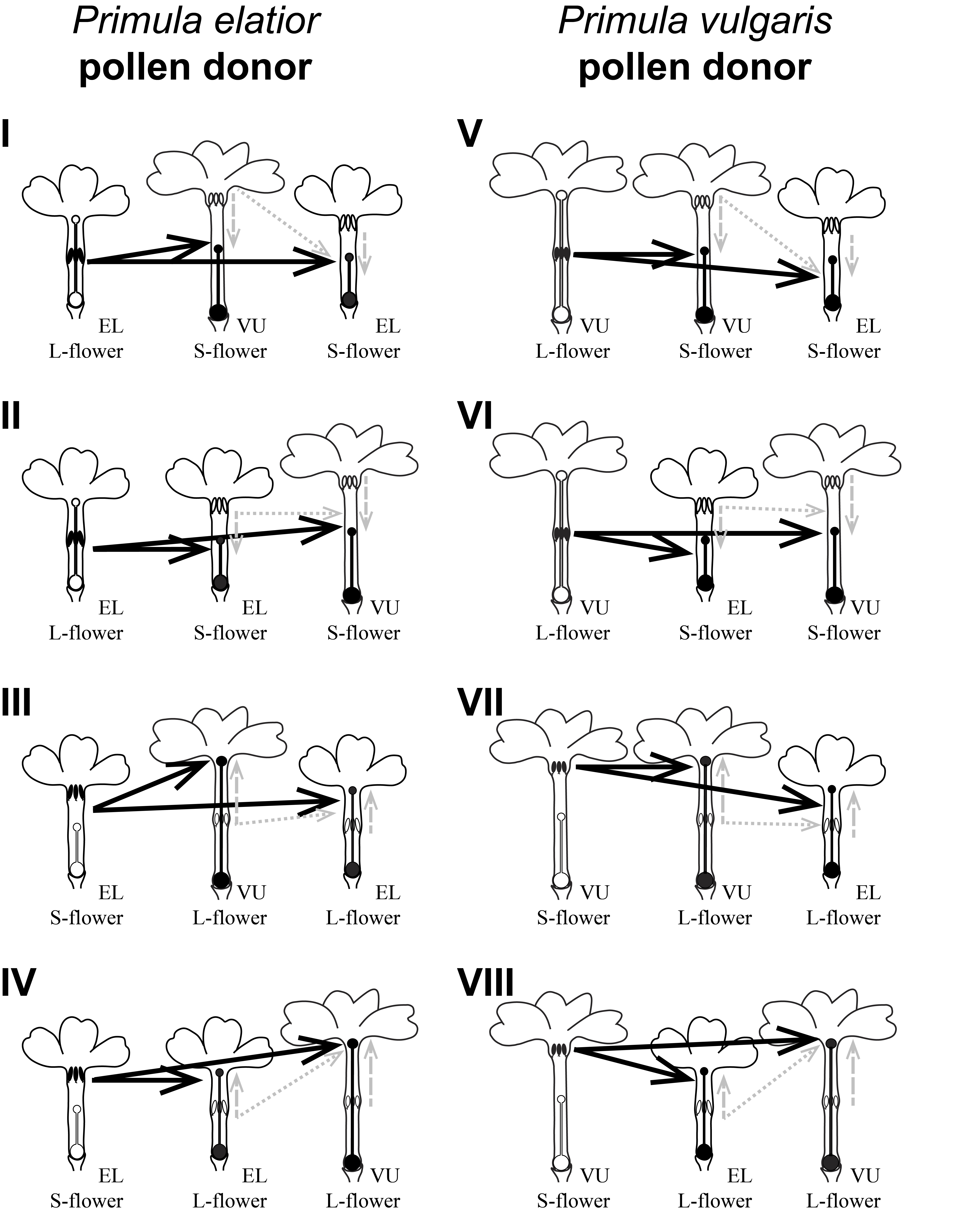


**Figure S2.** Mechanical isolation: experimental design used to compare intra- and interspecific pollen transfer between low and high reciprocal organs of *Primula elatior* and *P. vulgaris*. Roman numbers indicate triad numbers reported in Table 2. Transfer of pollen from pollen donors to recipient flowers is indicated by black solid lines (data used to calculate *RI*_mech_). Transfer of pollen within and between recipient flowers is indicated by grey lines (intramorph pollen: dotted lines; intra-floral pollen: dashed lines). Abbreviations: EL=*P. elatior*, VU=*P. vulgaris*, L=long-styled, S=short-styled.

**
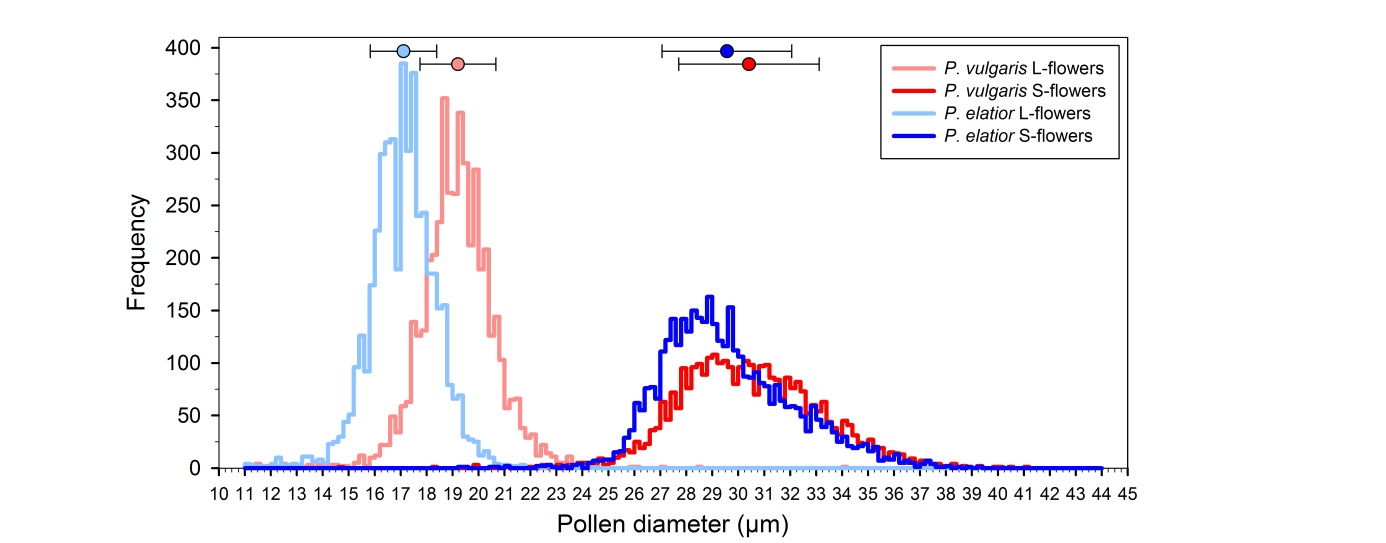
**

**Figure S3.** Mean diameters (circles) ± standard deviations and frequency distributions (lines) of pollen-grain sizes of long- (L-) and short-styled (S-) flowers of *Primula elatior* and *P. vulgaris*. We used ImageJ 1.40 (http://rsbweb.nih.gov/ij/) to measure pollen grains of 18 long-styled (4654 pollen grains) and 23 short-styled (3956 pollen grains) pollen donors of *P. elatior* and 18 long-styled (4512 pollen grains) and 24 short-styled (3326 pollen grains) pollen donors of *P. vulgaris*. Pollen grains from L-flowers of *P. elatior* and *P. vulgaris* had mean diameters of 17.11±1.28 µm (± standard deviation) and 19.20±1.46 µm, respectively, while S-flowers of *P. elatior* and *P. vulgaris* had mean diameters 29.56±2.5 µm and 30.41±2.70 µm, respectively. The difference between the L-morph and S-morph pollen-size class allowed us to differentiate between intermorph pollen *vs*. self- and intramorph pollen in the pollen-transfer experiment (see Table 2, Fig. S2).


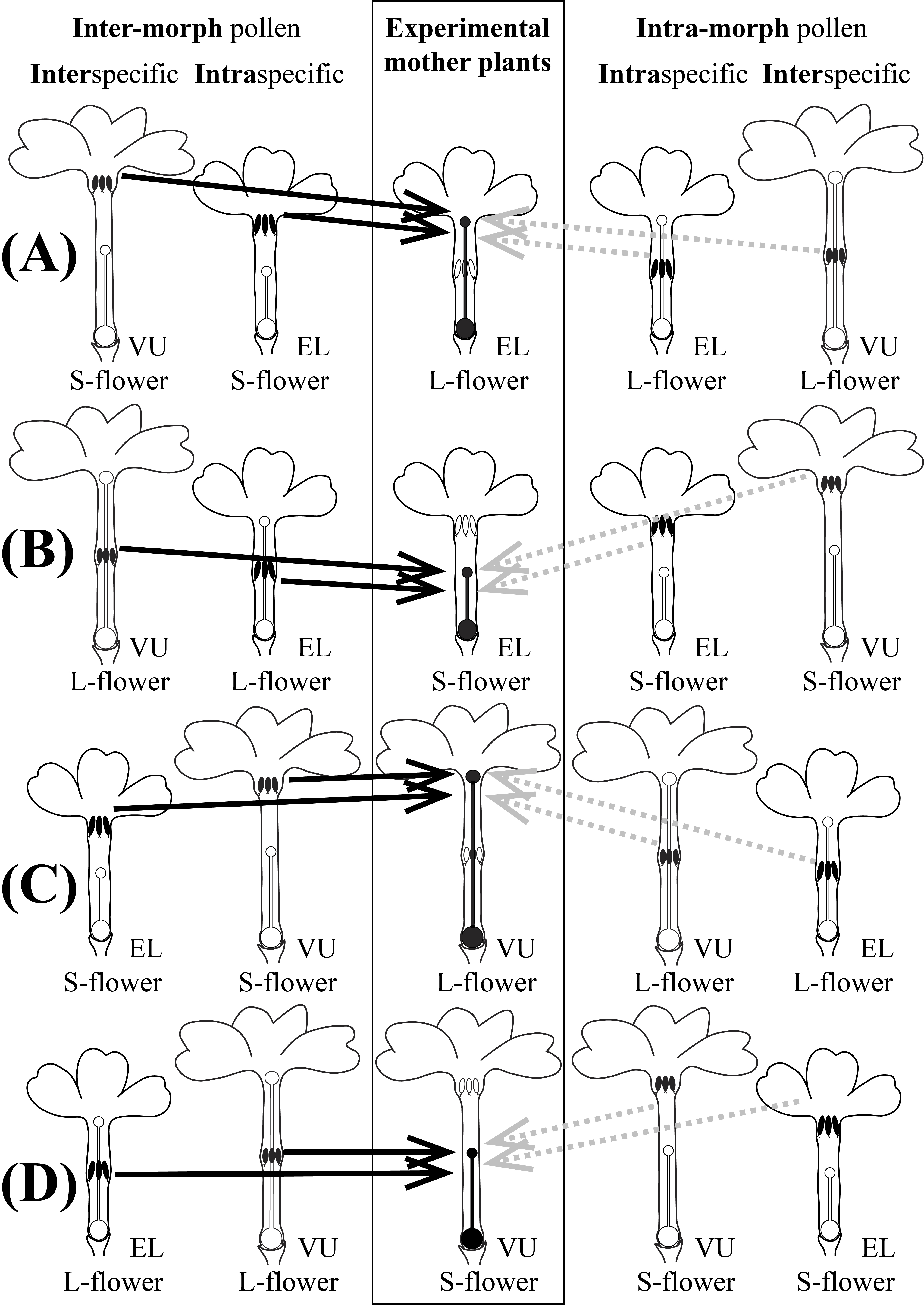


**Figure S4.** F1 seedling formation and interspecific intramorph incompatibility: Graphical representation of the four pollination treatments (*i.e.,* intermorph intraspecific, intermorph interspecific, intramorph intraspecific, and intramorph interspecific) performed on (A) long- and (B) short-styled plants of *Primula elatior* and (C) long-styled and (D) short-styled plants of *P. vulgaris*. Inter- and intraspecific pollinations between reciprocal flowers (intermorph) are indicated by black lines, the ones between non-reciprocal flowers (intramorph) by grey dotted lines. Abbreviations: EL=*P. elatior*, VU=*P. vulgaris*, L=long-styled, S=short-styled. Data from this experiment were used to quantify F1 seedling formation (*RI*_seedling_; intermorph crosses; see Table 3) and to assess strength of intramorph incompatibility in interspecific crosses (intramorph crosses).

**
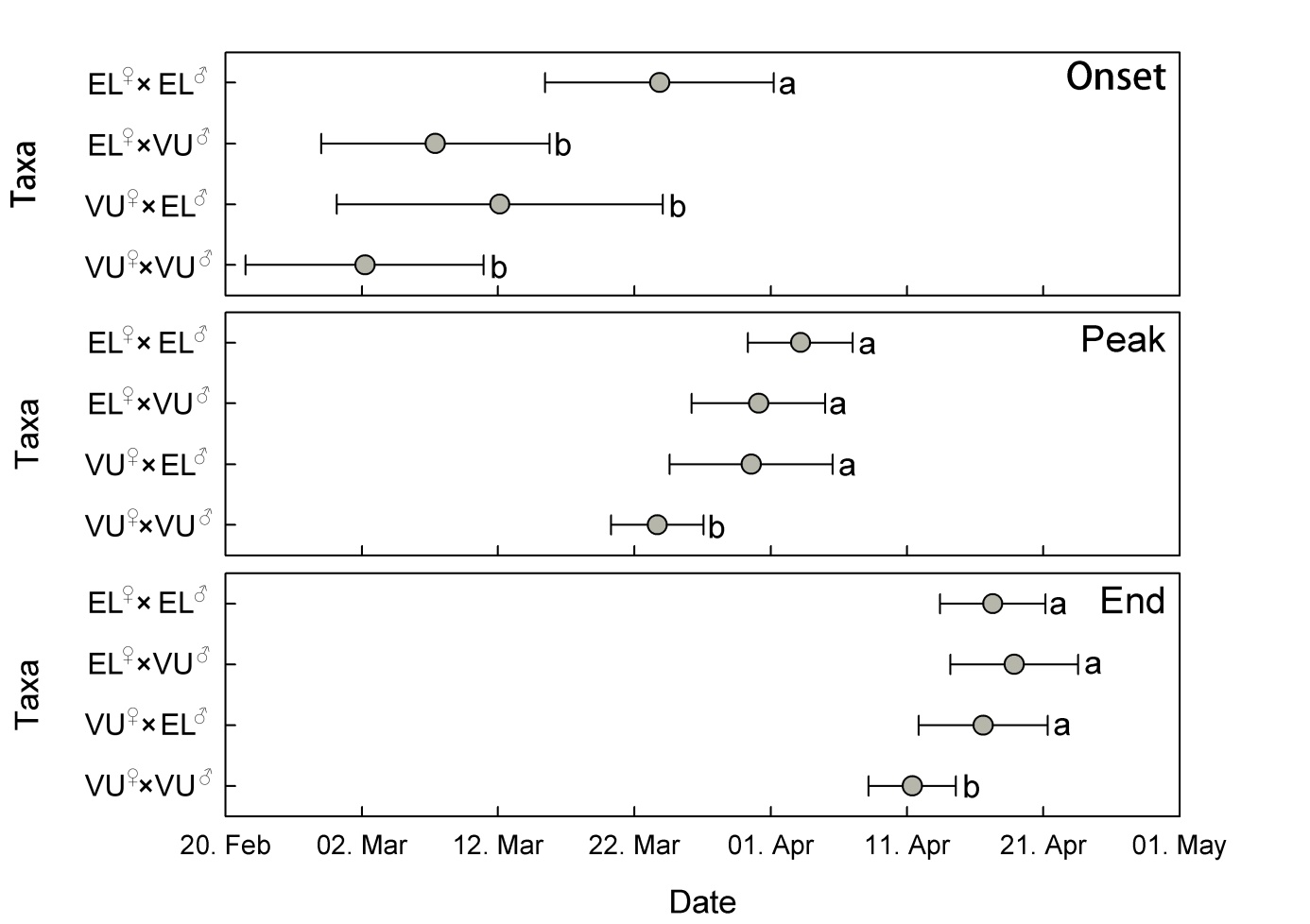
**

**Figure S5.** Flowering periods and phenological isolation: Means and standard deviations of onset (date when the first flower opens), peak (date when the maximal number of flowers is open), and end (date when the last flower withers) of the flowering time of *Primula elatior* (EL^♀^×EL^♂^), *P. vulgaris* (VU^♀^×VU^♂^), and their F1 hybrids (EL^♀^×VU^♂^ and VU^♀^×EL^♂^). Results based on a total of 20, 22, 17, and 15 plants from *P. elatior*, *P. vulgaris,* EL^♀^×VU^♂^, and VU^♀^×EL^♂^ hybrids, respectively, that survived winter and bloomed in spring. Kruskal-Wallis tests demonstrate that onset (*H* = 33.937, *P* < 0.001), peak (*H* = 36.380, *P* < 0.001), and end (*H* = 28.705, *P* < 0.001) of flowering time differed between parents and hybrids. Different letters indicate significantly different means of flowering dates in pairwise comparisons (significance level: *P* ≤ 0.05). Census data were used to calculate phenological isolation (*RI*_phenoP_) and F1 phenology (*RI*_phenoF1_; see text and Fig. 3): *RI*_phenoP_ for *P. elatior* was 0.237 and for *P. vulgaris* 0.417; mean *RI*_phenoF1_ for *P. elatior* was -0.046 (-0.040 and -0.053 [*RI*_EL♀×VU♂_ and *RI*_VU♀×EL♂_]) and for *P. vulgaris* -0.136 (-0.093 and -0.179; see Table 3).


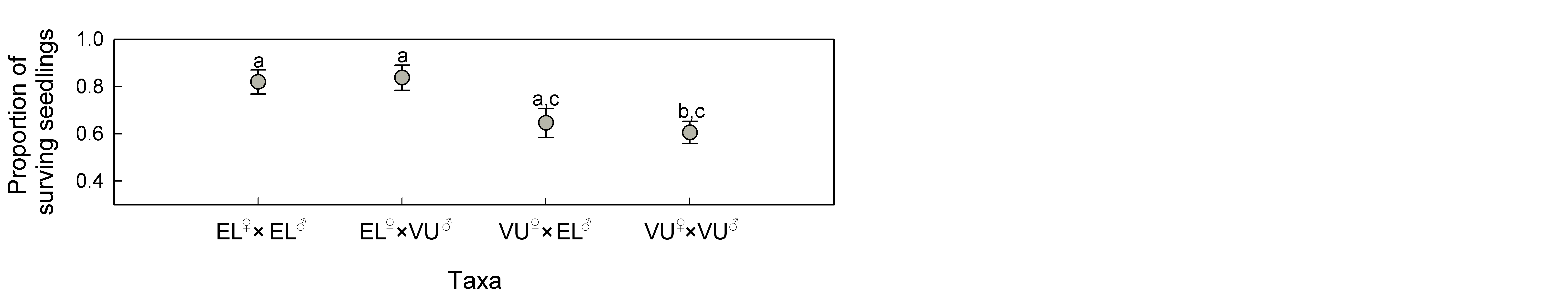


**Figure S6.** F1 survivorship: Mean values and standard errors (estimated from generalized linear mixed effects models) of proportion of seedlings that survived summer for *Primula elatior* (EL^♀^×EL^♂^), *P. vulgaris* (VU^♀^×VU^♂^), and F1 hybrids (EL^♀^×VU^♂^ and VU^♀^×EL^♂^). Generalized linear mixed effects models show that number of seedlings surviving to maturity significantly differed among *P. elatior*, *P. vulgaris*, EL^♀^×VU^♂^ hybrids, and VU^♀^×EL^♂^ hybrids (*F*_3,154_ = 3.882, *P* = 0.010). Different letters indicate significantly different means in pairwise comparisons (significance level: *P* ≤ 0.05). Mean values were used to calculate F1 survivorship (*RI*_survivorship_): mean *RI*_survivorship_ for *P. elatior* was 0.054 (-0.011and 0.118 [*RI*_EL♀×VU♂_ and *RI*_VU♀×EL♂_]) and for *P. vulgaris* -0.097 (-0.161 and -0.033; see Table 3).


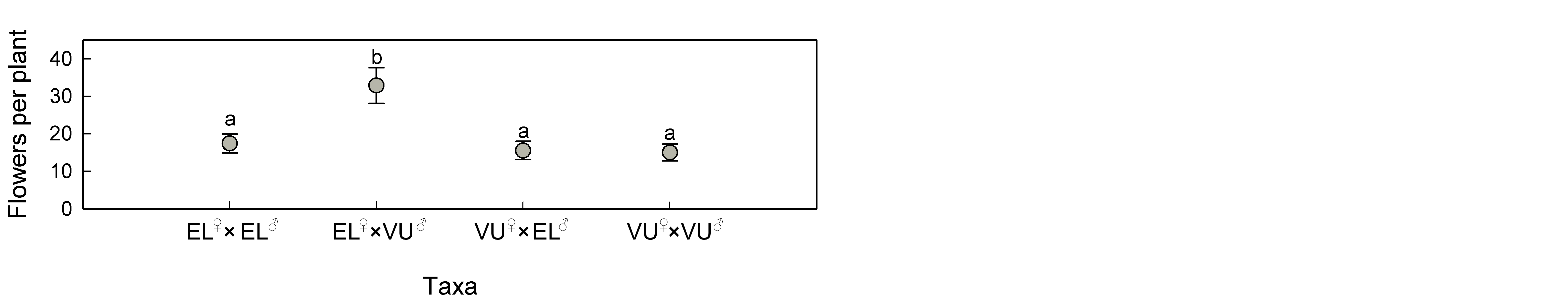


**Figure S7.** F1 flower production: Means and standard errors (estimated from generalized linear mixed effects models) of numbers of flowers produced during the entire blooming period of *Primula elatior* (EL^♀^×EL^♂^), *P. vulgaris* (VU^♀^×VU^♂^), and their F1 hybrids (EL^♀^×VU^♂^ and VU^♀^×EL^♂^). Results base on a total of 20, 22, 17, and 15 plants from *P. elatior*, *P. vulgaris,* EL^♀^×VU^♂^-, and VU^♀^×EL^♂^-hybrids, respectively, that survived winter and bloomed in spring 2014 (see Fig. S1). Generalized linear mixed effects models show that number of flowers differ significantly among *P. elatior*, *P. vulgaris*, EL^♀^×VU^♂^-hybrids, and VU^♀^×EL^♂^-hybrids (*F*_3,48_ = 29.877, *P* < 0.001). Different letters indicate significantly different means in pairwise comparisons (significance level: *P* ≤ 0.05). Mean values were used to calculate F1 flower production (*RI*_flower_): mean *RI*_flower_ for *P. elatior* was -0.125 (-0.307 and 0.057 [*RI*_EL♀×VU♂_ and *RI*_VU♀×EL♂_]) and for *P. vulgaris* -0.194 (-0.372 and -0.017; see Table 3).


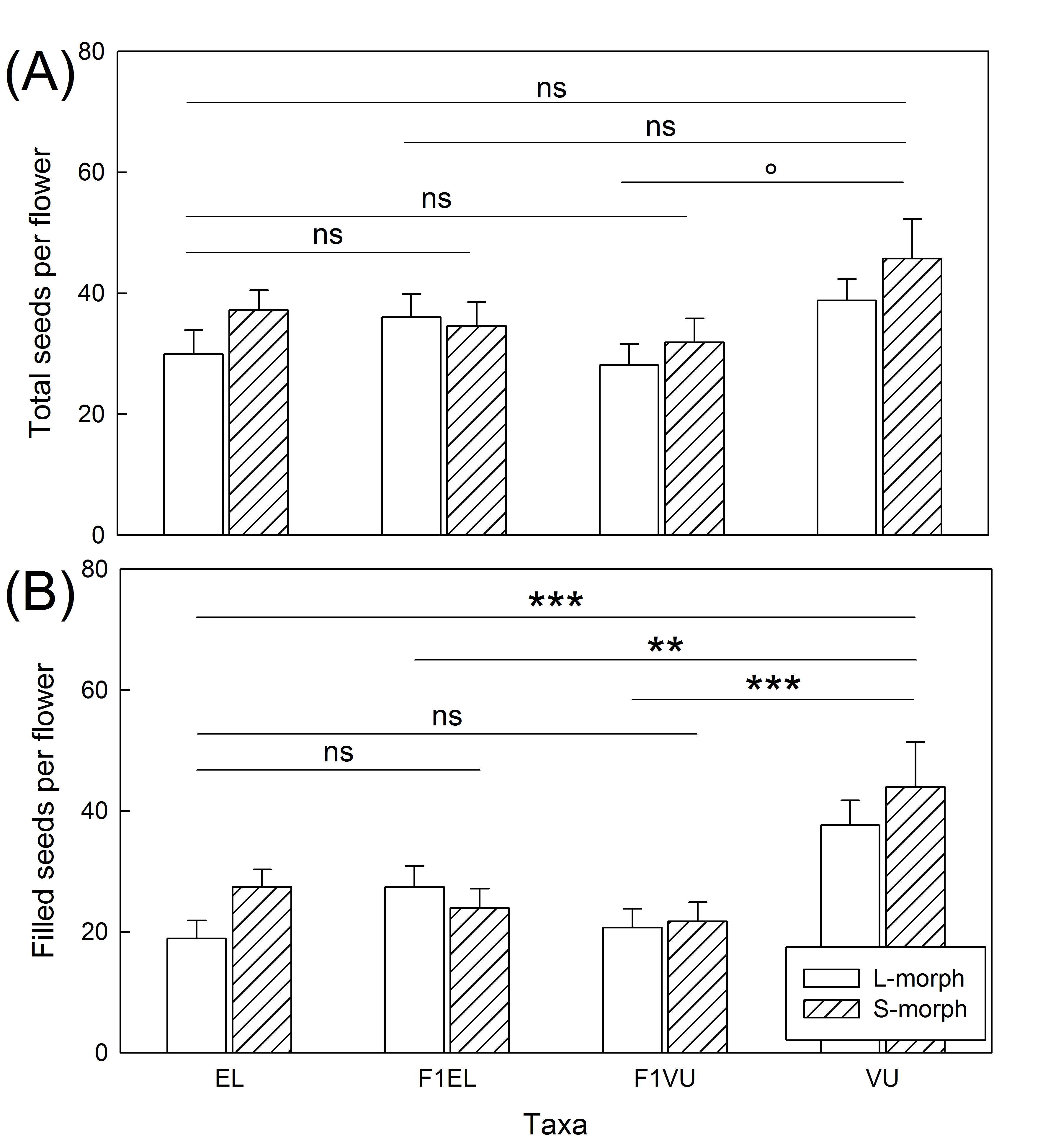


**Figure S8.** F1 seed set: Means and standard errors (estimated from generalized linear mixed effects models) of numbers of (A) total seeds and (B) filled seeds produced by long-styled (L-) and short-styled (S-) flowers of *Primula elatior* (EL), *P. vulgaris* (VU) and EL^♀^×VU^♂^ (F1EL) and VU^♀^×EL^♂^ hybrids (F1VU). Generalized linear mixed effects model show (marginally) significant effects of *plant class* (A: *F*_3,52_ = 2.745, *P* = 0.052; B: *F*_3,55_ = 8.500, *P* < 0.001), but no significant effect of *morph identity* (A: *F*_1,52_ = 1.980, *P* = 0.165; B: *F*_1,55_ = 1.241, *P* = 0.332) and interaction between *plant class* and *morph identity* (A: *F*_3,52_ = 0.485, *P* = 0.694; B: *F*_3,55_ = 1.266, *P* = 0.295). Significance levels: *P* ≤ 0.001 (***), *P* ≤ 0.01 (**), *P* = 0.051 (°), or not significantly different *P* > 0.05 (ns). Sequential Bonferroni correction was implemented to account for multiple tests. Mean values of number of filled seeds were used to calculate F1 seed set (*RI*_seed set_): mean *RI*_seed set_ for *P. elatior* was -0.116 (-0.185 and -0.047 [*RI*_EL♀×VU♂_ and *RI*_VU♀×EL♂_]) and 0.092 (0.068 and 0.116) in L- and S-flowers, respectively; mean *RI*_seed set_ for *P. vulgaris* was 0.223 (0.157 and 0.290) and 0.317 (0.296 and 0.339) in L- and S-flowers, respectively (see Table 3).


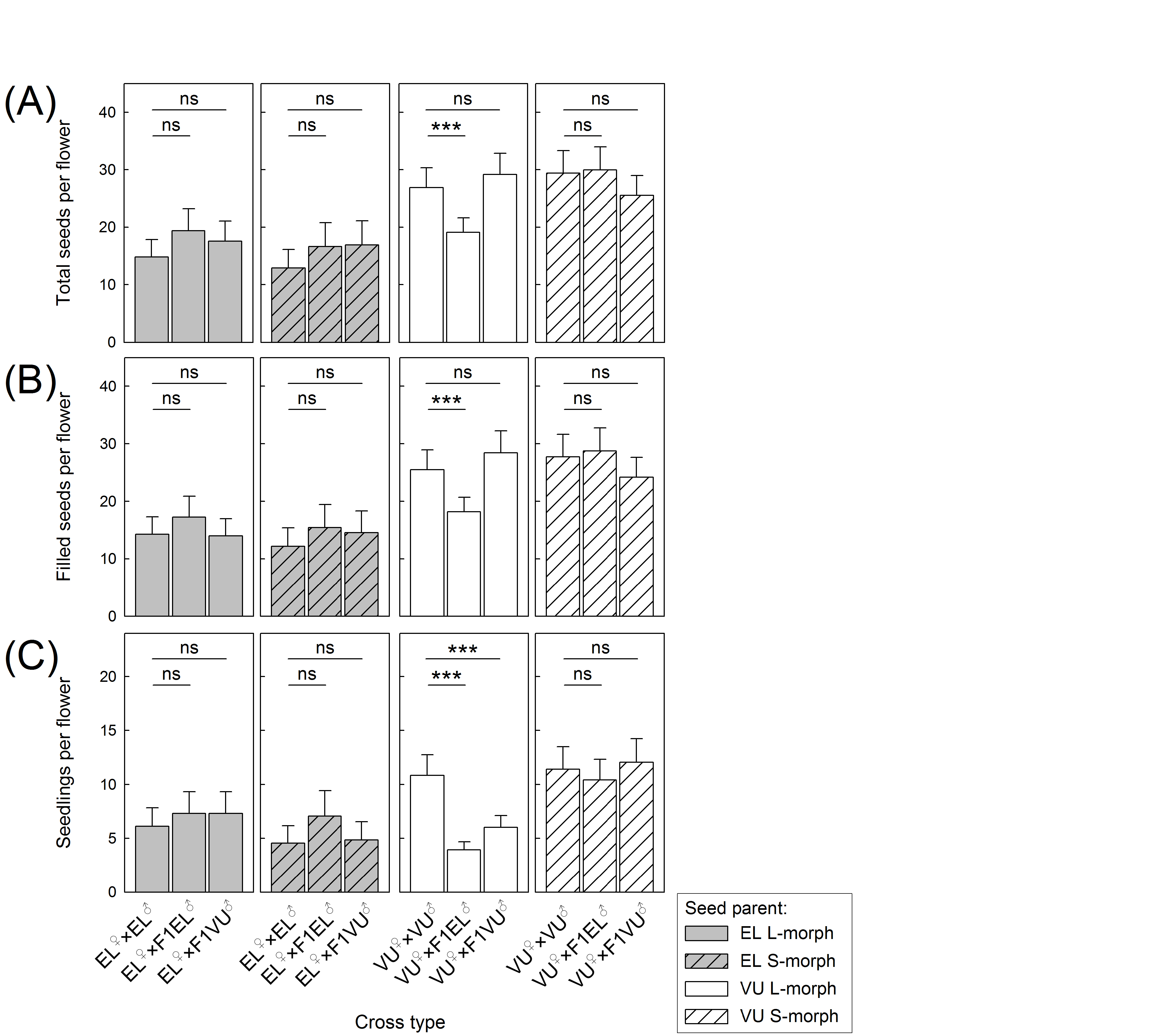


**Figure S9.** F1 male sterility: Means and standard errors (estimated from generalized linear mixed effects models) of number of (A) total seeds, (B) filled seeds, and (C) seedlings per flower produced by long- (L) and short-styled (S) morphs of *Primula elatior* (EL) and *P. vulgaris* (VU) pollinated with intermorph pollen of the same species (EL^♀^×EL^♂^ and VU^♀^×VU^♂^, respectively), pollen of EL^♀^×VU^♂^ hybrids (EL^♀^×F1EL^♂^ and VU^♀^×F1EL^♂^, respectively), and pollen of VU^♀^×EL^♂^ hybrids (EL^♀^×F1VU^♂^ and VU^♀^×F1VU^♂^, respectively). Generalized linear mixed effects model show significant effect of *pollination treatment* (A: *F*_5,144_ = 6.454, *P* ≤ 0.001; B: *F*_5,144_ = 5.624, *P* ≤ 0.001, C: *F*_5,144_ = 11.959, *P* ≤ 0.001) and *pollination treatment* × *morph identity* (A: *F*_5,144_ = 8.998, *P* ≤ 0.001; B: *F*_5,144_ = 9.889, *P* ≤ 0.001, C: *F*_5,144_ = 9.245, *P* ≤ 0.001), but no significant effect of *morph identity* (A: *F*_1,46_ = 0.006, *P* = 0.937; B: *F*_1,45_ = 0.018, *P* = 0.894, C: *F*_1,43_ = 0.463, *P* = 0.500). Significance levels: *P* ≤ 0.001 (***) or not significantly different *P* > 0.05(ns). Sequential Bonferroni correction was implemented to account for multiple tests. Mean values of number of seedlings were used to calculate F1male sterility (*RI*_male_): mean *RI*_male_ for *P. elatior* was -0.088 (-0.088 and -0.088 [*RI*_EL♀×VU♂_ and *RI*_VU♀×EL♂_]) and -0.123 (-0.215 and -0.031) in L- and S-flowers, respectively; mean *RI*_male_ for *P. vulgaris* was 0.378 (0.469 and 0.287) and 0.008 (0.044 and -0.028) in L- and S-flowers, respectively (see Table 3).

**Literature**

Keller, B., J. M. de Vos, and E. Conti. 2012. Decrease of sexual organ reciprocity between heterostylous primrose species, with possible functional and evolutionary implications. Ann Bot 110:1233-1244.

Richards, J. H. and S. Koptur. 1993. Floral variation and distyly in *Guettarda scabra* (Rubiaceae). Am. J. Bot. 80:31-40.
